# The use of chicken and insect infection models to assess the virulence of African *Salmonella* Typhimurium ST313

**DOI:** 10.1371/journal.pntd.0007540

**Published:** 2019-07-26

**Authors:** Lizeth Lacharme-Lora, Siân V. Owen, Richard Blundell, Rocío Canals, Nicolas Wenner, Blanca Perez-Sepulveda, Wai Yee Fong, Rachel Gilroy, Paul Wigley, Jay C. D. Hinton

**Affiliations:** 1 Department of Functional and Comparative Genomics, Institute of Integrative Biology, University of Liverpool, Liverpool, United Kingdom; 2 Department of Biomedical Informatics, Harvard Medical School, Boston, Massachusetts, United States of America; 3 Department of Veterinary Pathology and Public Health, Institute of Veterinary Science, University of Liverpool, United Kingdom; 4 Department of Infection Biology, Institute of Infection and Global Health, University of Liverpool, Liverpool, United Kingdom; University of Colorado Health Sciences Center, UNITED STATES

## Abstract

Over recent decades, *Salmonella* infection research has predominantly relied on murine infection models. However, in many cases the infection phenotypes of *Salmonella* pathovars in mice do not recapitulate human disease. For example, *Salmonella* Typhimurium ST313 is associated with enhanced invasive infection of immunocompromised people in Africa, but infection of mice and other animal models with ST313 have not consistently reproduced this invasive phenotype. The introduction of alternative infection models could help to improve the quality and reproducibility of pathogenesis research by facilitating larger-scale experiments. To investigate the virulence of *S*. Typhimurium ST313 in comparison with ST19, a combination of avian and insect disease models were used. We performed experimental infections in five lines of inbred and one line of outbred chickens, as well as in the alternative chick embryo and *Galleria mellonella* wax moth larvae models. This extensive set of experiments identified broadly similar patterns of disease caused by the African and global pathovariants of *Salmonella* Typhimurium in the chicken, the chicken embryo and insect models. A comprehensive analysis of all the chicken infection experiments revealed that the African ST313 isolate D23580 had a subtle phenotype of reduced levels of organ colonisation in inbred chickens, relative to ST19 strain 4/74. ST313 isolate D23580 also caused reduced mortality in chicken embryos and insect larvae, when compared with ST19 4/74. We conclude that these three infection models do not reproduce the characteristics of the systemic disease caused by *S*. Typhimurium ST313 in humans.

## Author summary

*Salmonella* Typhimurium ST313 is associated with systemic infection in human populations in sub-Saharan Africa, and contrasts with the related pathovariant ST19 which causes gastrointestinal disease worldwide. Although the systemic pathology associated with ST313 infection in humans has been comprehensively documented in clinical and epidemiological studies, such pathology has been inconsistently reproduced in animal models of infection. Animal models that reliably recapitulate ST313 infection in humans are needed to study the biological mechanisms underpinning the systemic disease caused by ST313. In this study we performed extensive infection experiments, using several defined and alternative animal infection models to look for robust phenotypes that differentiate infection by *S*. Typhimurium ST313 from ST19. Large sample sizes and multivariate statistical analysis of infection data for inbred chicken lines allowed us to detect small but consistent differences between the strains. Overall, *S*. Typhimurium ST313 was associated with a reduced infection burden and pathology relative to ST19. This subtle phenotype may reflect the limitation of animal models to accurately represent infection by pathogens that have adapted to specific host phenotypes, for example, immunodeficiency in humans. Our study demonstrates the challenge of using animal models to differentiate closely-related bacterial pathovariants, and shows that inter-pathovar differences detected in animal models of infection often do not reflect clinical differences in humans at the level of disease mechanism.

## Introduction

*Salmonella* Typhimurium sequence type (ST) 313 is a novel pathovariant circulating in sub-Saharan Africa that causes invasive non-typhoidal Salmonella (iNTS) disease [1]. The most common *S*. Typhimurium ST present throughout the rest of the world is ST19, which generally causes gastroenteritis. Recently, we reported the use of a comparative transcriptomic approach to identify genes that were differentially expressed between ST313 and ST19 in a variety of environmental conditions and during macrophage infection [2].

Previously, we used the chicken model to study the infection biology of ST313 and ST19 *S*. Typhimurium strains. The data showed that *S*. Typhimurium ST313 strains caused invasive disease in Lohmann brown chickens and caused a more invasive phenotype than ST19 strains at early stages of infection [3]. However, the research was limited by small sample sizes and the exclusive use of outbred chicken lines.

In *Salmonella* research, the majority of infection studies have been carried out with inbred mouse lines such as BALB/c and C57BL/6 [4,5]. Other animal models such as pigs, rhesus macaques and calves have also been used, but results with *S*. Typhimurium ST313 have been inconsistent [6–8]. In some models, like the chicken, mouse, bovine and non-human primate models, ST313 strains were capable of invading the intestinal mucosa and eliciting intestinal inflammation [3,6]. In other models, such as in streptomycin-treated mice and bovine ligated ileal loops, ST313 strains had a slightly reduced ability to cause intestinal inflammation in comparison to ST19 [9]. A recent review article suggested that these differences may be attributed to variations between the animal models that were involved and the different ST19 strains that were used as the comparator [10].

Fewer studies have been done in chickens, and outbred lines (which represent a genetically heterologous population) such as Lohmann Brown laying chickens, have most commonly been used. However, several inbred chicken lines (representing populations of genetically-identical animals) have been used for *Salmonella* infection research [11–13]. Different inbred lines vary in their resistance to systemic salmonellosis caused by *S*. Typhimurium ST19, largely due to the salmonellosis resistance locus *SAL1* which is associated with an increased pro-inflammatory response [14]. Inbred lines previously used in *Salmonella* studies include the White Leghorn-derived inbred lines W and 6_1_, which show a *“Salmonella-resistant”* phenotype; and lines 7_2_, Cb4 and 15, which show a *“Salmonella-susceptible”* phenotype.

This *Salmonella* resistance classification was based on infection studies performed in 1-day-old chicks, infected with *S*. Typhimurium ST19 strain F98, and in older birds with the avian-adapted serovar *Salmonella* Gallinarum [11]. The resistant phenotype manifests as reduced mortality, tolerance to a higher infectious dose and reduced systemic pathology. However, the same birds showed a weak resistance phenotype after oral infection with *S*. Typhimurium ST19 at 2 weeks old, whilst retaining a strong resistant phenotype to *S*. Gallinarum [11].

Reduced biological bird-to-bird variance has been observed with inbred lines of chickens compared with outbred populations, improving the quality of the experimental outcomes [15]. Other less commonly-used infection models such as the chicken embryo [16] and the wax moth larvae (*G*. *mellonella*) models [17] are particularly experimentally-tractable and reliable, and therefore show promise for use in *Salmonella* pathogenesis research [18].

We investigated the relative virulence of *S*. Typhimurium ST313 isolate D23580 and ST19 isolate 4/74 in outbred and inbred chicken lines, chick embryos and *G*. *mellonella* larvae.

## Materials and methods

### Ethics statement

All the procedures were performed in accordance with UK legislation governing experimental animals under project licence PPL40/3652 and the experimental protocols were approved by the University of Liverpool ethical review process.

### Bacterial strains and growth conditions

*S*. Typhimurium strain 4/74, a representative strain of nontyphoidal *Salmonella* sequence type 19 (ST19), D23580, a representative strain of nontyphoidal *Salmonella* sequence type 313 (ST313), and derivates from these strains (S1 and S2 Tables) were used in this study. Strain D23580 was isolated from an HIV^−^ child from Malawi with bloodstream infection and use of this strain has been approved by the Malawian College of Medicine (COMREC ethics no. P.08/14/1614). All isolates were maintained as frozen stocks at −80°C. Prior to infection, isolates were grown in LB (Lennox) broth at 37°C in a shaking incubator at 220 rpm for 18 h.

### Outbred chickens

One-day-old Lohmann Brown outbred chickens of mixed sex were obtained from a commercial hatchery. 48 birds were randomly divided into two groups, one was inoculated with *S*. Typhimurium strain 4/74 and the other group was inoculated with *S*. Typhimurium strain D23580.

### Inbred chicken lines

One-day-old specific pathogen-free White Leghorn-derived inbred lines W and 6_1_ chickens (*“Salmonella-resistant”*), and lines 7_2_, Cb4 and 15 chickens (*“Salmonella-susceptible”*) were supplied by the National Avian Research Facility (Roslin Institute, Edinburgh). Between 28 and 40 birds of mixed sex were used per line. For each line, birds were randomly separated in to two groups as above.

### Experimental infection of chickens

Both inbred and outbred chicks were housed in the University of Liverpool high-biosecurity poultry unit. Birds were housed in accommodation meeting the UK legislation requirements and were given *ad libitum* access to water and a vegetable protein-based pelleted diet (Special Diet Services, Witham, Essex, UK). Chicks were housed on wood shavings in floor pens at a temperature of 30°C. All housing and environmental conditions were identical between groups. The birds were tagged with metal wing bands to allow identification of individuals. All animals were checked a minimum of twice daily to ensure their health and welfare. Prior to experimental infection, all birds were confirmed as *Salmonella*-free by cloacal swabs, which were streaked onto selective XLT4 (Lab M) and grown for 24 h at 37°C.

For both sets of birds (outbred and inbred lines), individual groups were inoculated by oral gavage with 10^8^ CFU of either *S*. Typhimurium 4/74 or D23580 in 0.2 ml LB (Lennox) broth (overnight cultures) when the birds were two weeks old. For inbred birds, one further group of birds was mock-infected with sterile Phosphate Buffered Saline (PBS) as a control for histopathological studies.

At 3, 7 and 12 days post-challenge, 4–7 birds from each group were culled by cervical dislocation and post-mortem examinations were carried out. Assessment of *Salmonella* colonisation levels and histopathological studies were carried out.

### Assessment of *Salmonella* colonisation levels in the chicken model

Samples from spleen, liver and caecal content were removed aseptically from each bird, weighed and diluted 1:5 (wt/vol) in sterile PBS. Tissues were then homogenized in a Colworth 80 microstomacher (A.J. Seward & Co. Ltd). Serial 10-fold dilutions were made of each sample in PBS, and according to the method of Miles and Misra [19], triplicate 20 μl spots were plated onto Harlequin™ *Salmonella* ABC Medium (Lab M). The plates were incubated at 37°C for 24 h, and colonies were enumerated to give CFU/g of sample.

### Histopathological studies

Liver, spleen and caecal tissue samples from inoculated birds were taken at each time point and fixed in 4% paraformaldehyde in PBS. Tissues were embedded in paraffin wax, cut and stained with haematoxylin and eosin, and then examined by a board certified veterinary pathologist at the University of Liverpool. All sections were examined blind and were scored individually for each tissue from each animal based on the scoring system detailed in Parsons *et al*, 2013 [3], to make our data comparable to the data previously published.

### Assessment of *Salmonella* colonisation levels in the chick embryo model

Fertilised White Leghorn chicken eggs were obtained from Lees Lane Poultry, Wirral, or Tom Barron, Preston, UK. Fertilised eggs were incubated at 37°C with a relative humidity of 50–60% (Octagon 40 egg turner incubator, Brinsea, Weston Super Mare, UK) until E11 (embryonic day 11) or E14 and all animal work followed UK regulations. On the 10th day of embryonic development the eggs were candled to detect and discard those that were not fertilized or had dead embryos. On day E11 a hole was aseptically made in the egg shell and 10^2^ CFU bacterial suspensions from an overnight culture diluted in PBS were inoculated intra-allantoically using a sterile 1 ml syringe with a 27G needle attached. The hole was then covered with tape and the eggs returned to the incubator. Injections with PBS and *S*. Typhimurium 4/74 and D23580 Δ*rpoE*::*frt* mutants were used as negative controls. At the end of the incubation period, embryos were separated from the eggs and washed with sterile PBS several times. Embryos were dissected and their livers were extracted, washed several times with PBS and placed on ice. The number of bacteria in the liver was determined using the same protocol used in the chicken model. Yolk samples were taken prior to extraction of the embryos.

### Competition experiments in the chick embryo model

For the competition assay, 11-day-old embryos were inoculated via the allantoic cavity with a mix of equal numbers of *S*. Typhimurium D23580 Tc^R^ (JH3950, S1 Table) and 4/74 Km^R^ (JH4284, S1 Table) in 100 μl of PBS. The combined bacterial inoculum was ~ 4 × 10^2^ CFU. The numbers of each strain in the inoculum and in the liver and yolk of inoculated embryos were calculated by plating onto LB (Lennox) containing 25 μg/ml tetracycline or 50 μg/ml kanamycin. The competitive index was calculated as the ratio of D23580 and 4/74 at 16 hours post-inoculation, divided by the same ratio in the inoculum.

### Construction of wild-type strains carrying a selection marker

The *S*. Typhimurium 4/74 *ΔSL1483*::*aph* mutant (JH4284) (S1 and S2 Tables) was constructed with the sole purpose of having a wild-type strain with a selection marker, in this case resistance to kanamycin. The resistance marker was introduced by λ Red recombination in a pseudogene (*SL1483*) that does not code for a functional protein. Briefly, the kanamycin resistance cassette (*aph*) was amplified from plasmid pKD4 using the oligonucleotides ins_STnc230_rev and del_SL1483_for. The resulting PCR product was transformed into 4/74 carrying the recombineering plasmid pSIM5-*tet* [20] by electroporation. The Δ*SL1483*::*aph* was transduced into 4/74 WT using the high-frequency-transducing bacteriophage P22 HT 105/1 *int-201* [21].

For D23580, a tetracycline resistant version of D23580 (D23580 Tc^R^), that carries the *tetRA* genes between the pseudogene *STMMW_41451* and the gene *STMMW_41461* (between coordinates 4441510 and 4441511 in D23580) was constructed by λ Red recombination as follows: the *tetRA* resistance cassette was amplified by PCR from the Tn*10* transposon with primers NW_202 and NW_203. The resulting PCR fragment was electroporated into D23580 wild-type carrying the λ Red recombination plasmid pKD46-Gm, as described previously [22], and tetracycline resistant recombinants were selected on LB agar plates supplemented with tetracycline hydrochloride (25 μg/ ml). The *tetRA* cassette was subsequently transduced into D23580 wild-type, using phage P22 HT 105/1 *int*-201 as previously described [23]. Both mutants were confirmed by sequencing with external primers.

### Assessment of mortality in the chicken embryo model

Groups of 10 eggs/strain were used. Eggs were inoculated at embryonic day 11 as above and incubated at 37°C for 24 hours. Embryo viability was recorded by candling and the mortality rate was calculated as the mean percentage of embryonic deaths at 24 hours post-infection.

### Assessment of mortality in the *G*. *mellonella* (wax moth larva) model

Genetically homogeneous final-instar greater wax moth *G*. *mellonella* larvae were purchased from BioSystems Technology UK. Groups of 10 larvae were contained in a petri dish lined with a circle of Whatman filter paper #1 and injected with 10 μl of bacterial suspension using a Hamilton 701LT syringe fitted with a 27G mm needle. All injections were administered via the last left pro-leg into the haemocoel. As controls, a group of larvae were injected with PBS and another group was not injected. The plates containing the larvae were incubated at 37°C for 24 hours at which point mortality rate was recorded. Larvae that did not show any movement in response to touch were considered dead.

### Statistical analysis

For the chicken model, a Mann-Whitney test was used to determine the statistical significance of differences between the strains in individual timepoints and tissue types, as the distribution was not normal.

To detect broader trends in the colonisation level and histopathological score data, we used R version 3.5.2 to construct seven linear models to predict tissue burden or histopathology as a function of strain, tissue type, chicken line and timepoint. For the outbred chicken data, the model contained 141 CFU/g tissue (log_10_) counts from three tissue types (caeca, liver and spleen) and 3 timepoints post infection (1, 3 and 5 days) (raw data available in S3 Table). For the inbred chicken data, the models contained 534 CFU/g tissue (log_10_) counts and 521 histopathological scores respectively, as well as both tissue count and histopathology score datasets split into the *Salmonella-susceptible* (7_2_, Cb4 and 15I) and *Salmonella-resistant* (W and 6_1_) chicken lines, The models included a total of 9 variables representing strain (n = 1), tissue (n = 2), chicken line (n = 4) and timepoint (n = 2). We used the car [24] package to determine significance of model parameters using type III sums of squares [25]. Linear model and Anova summary tables are shown in S4–S6 and S9–S11 Tables. For the chick embryo and *G*. *mellonella* larvae models, a t-test was used to determine the statistical significance of strain differences. The raw data from all experiments undertaken in this study can be found in S1 Dataset.

## Results

### Similar levels of virulence were observed for *S*. Typhimurium D23580 and 4/74 in outbred chickens

**Fig 1:**
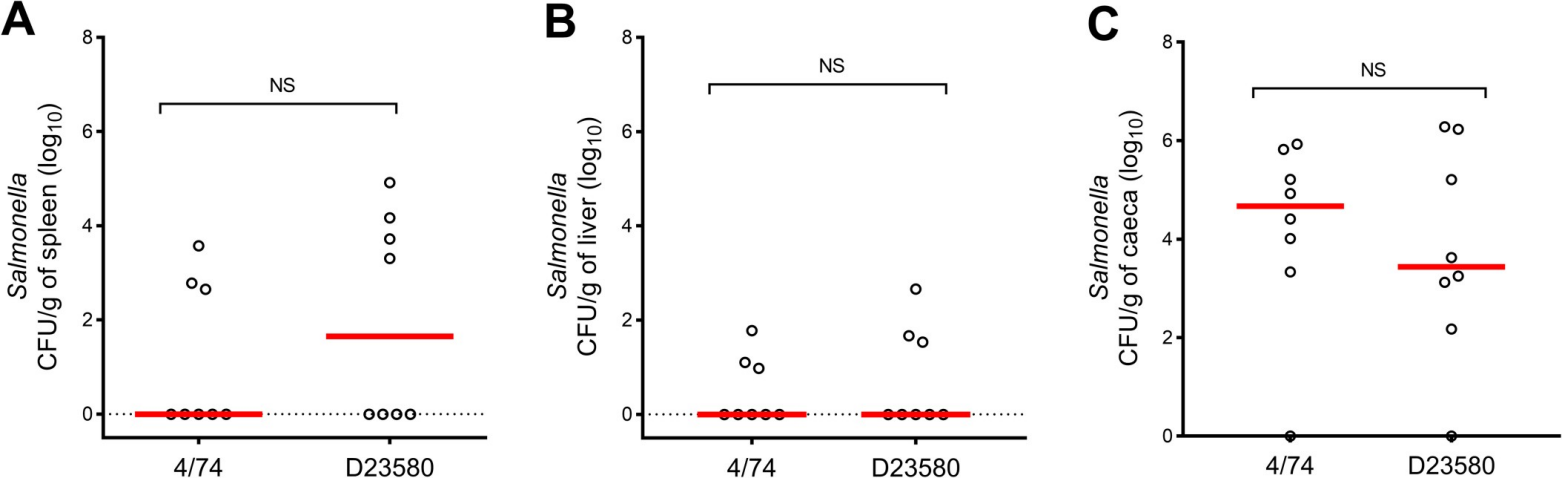
Similar levels of *S*. Typhimurium 4/74 and D23580 colonise outbred chickens. Viable counts (log_10_ CFU/g) of *S*. Typhimurium 4/74 and D23580 in spleen (A), liver (B) and caecal content (C) of outbred chickens are shown. *S*. Typhimurium 4/74 and D23580 at 3 days post-oral infection (10^8^ CFU) of 14-day-old Lohmann Brown Layers. Counts are shown for individual birds with the bar representing the median value (eight birds per strain). Statistical comparison was made using a Mann-Whitney test. NS: not significant, P > 0.05.

To study the ability of isolates D23580 and 4/74 to colonise chickens, we inoculated 14-day-old outbred Lohmann Brown Layers with 10^8^ bacteria by oral gavage. We sampled the caeca, liver and spleen of infected animals at 1, 3 and 5 days post-infection (dpi). The experiment was comparable to Parsons *et al*. 2013 [3], but used four times more animals per group. Fig 1 shows the colonisation levels at 3 dpi, indicating that the colonisation levels for the two strains were not significantly different when the experiment involved a large sample size (P > 0.05). Colonisation levels at 1 and 5 dpi were also assessed and showed no significant differences between strains (S1 Fig).

The abundance of bacteria in the liver, spleen and caeca of birds infected with strain D23580 were similar to those published by Parsons *et al*., 2013 [3] at all timepoints. However, the abundance of bacteria found in birds infected with 4/74 were somewhat different here, with lower levels found in caeca and higher levels found in spleen at 3 dpi compared to the previous study [3]. Linear modelling of the total dataset from the infection of outbred chickens with strains D23580 and 4/74 did not find bacterial strain to be a significant variable that affected the burden of infection (S3 Table).

### *S*. Typhimurium D23580 showed a lower infection burden in inbred chicken lines than 4/74

Animal models have been used in biomedical research for centuries. Beginning in the Pasteur era, infection studies involved outbred animals (genetically heterogeneous). However, large animal-to-animal variations prompted researchers to gradually move to inbred lines, as it became evident that genetic factors determined many animal characteristics. For example, genetically-homogeneous inbred mice yield more reproducible infections than outbred mice [26].

Since the results from our experimental infection of outbred birds (S1 Fig and S3 Table) did not show a differential phenotype for ST313 and ST19 strains [3], we hypothesized that this finding could reflect the bird-to-bird variation associated with outbred animals. Therefore, we compared the virulence of *S*. Typhimurium D23580 and 4/74 in inbred chicken lines.

**Fig 2:**
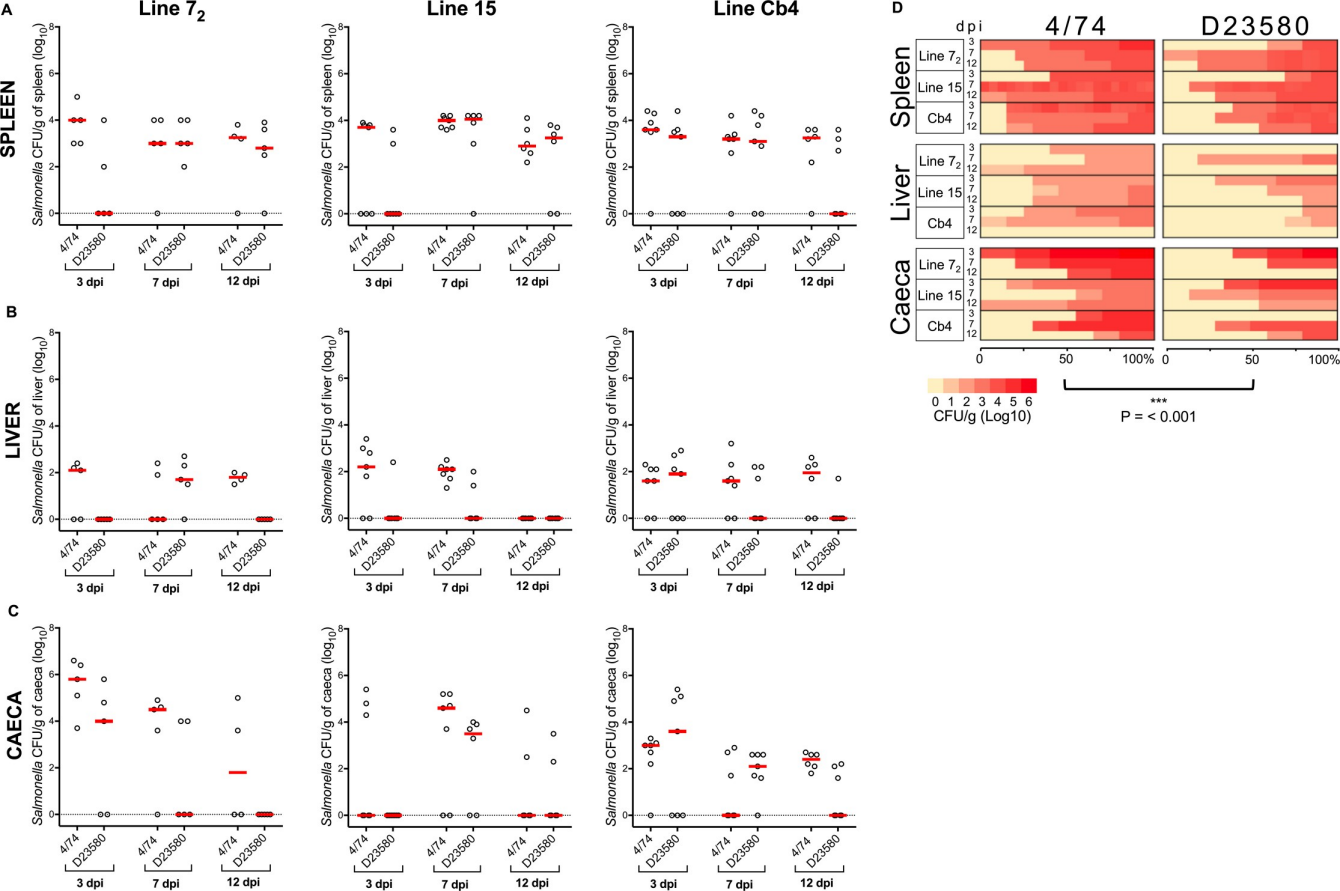
*S*. Typhimurium 4/74 is associated with an overall increased tissue burden in “*Salmonella-susceptible”* inbred chickens relative to D23580. 14-day-old lines 7_2_, 15 and Cb4 White Leghorn chickens were infected with 10^8^ CFU of *S*. Typhimurium 4/74 or D23580 and assessed at 3, 7 and 12 days post-oral infection. Each group contained four to seven birds. Statistical comparison was made using a Mann-Whitney test. NS: not significant, P > 0.05. Viable counts (CFU/g) of *S*. Typhimurium D23580 and 4/74 in spleen (A), liver (B) and caecal content (C) are shown. Counts are shown for individual birds with the bar representing the median value. A heatmap summarises the bacterial burden in all birds inoculated with 4/74 or D23580 (D). The heatmaps represents bacterial burden as a yellow (0 log_10_ CFU/g) to red (6 log_10_ CFU/g) colour scale and data are shown as percent of infected birds. Statistical modelling of the *“Salmonella-susceptible”* inbred chicken infection data showed that 4/74 was associated with an average increased tissue burden of 0.9 log_10_ CFU/g tissue (P < 0.001).

We first compared the two isolates in the inbred chicken lines 7_2_, 15 and Cb4 that had been reported to be *“Salmonella-susceptible”*. In all cases, the same infection procedure was used: oral-gavage of 14-day-old birds with 10^8^ cells and quantification of bacterial burden in the spleen, liver and caeca at 3, 7 and 12 dpi. In all three *“Salmonella-susceptible”* lines, some statistically significant differences (p-value < 0.05) were seen in pairwise comparisons between strains D23580 and 4/74 at 3, 7 and 12 dpi. Specifically, higher levels of colonisation of caeca, liver and spleen were seen in birds inoculated with 4/74 compared to birds inoculated with D23580. Specifically more 4/74 than D23580 bacteria were isolated from spleen at 3 dpi (P = 0.047) and liver at 12 dpi (P = 0.007) for Line 7_2_; from liver at 7 dpi (P = 0.018) for Line 15; and from the caeca of Line Cb4 at 12 dpi (P = 0.010, Fig 2).

To detect broader trends in infectivity between the strains, we used a linear model to predict log_10_CFU/g tissue as a function of time point, chicken line, strain and tissue. In the susceptible chicken lines, strain 4/74 was associated with a small, but consistent increase in tissue burden relative to D23580 (average increase of 0.9 log_10_ CFU/g tissue, P < 0.001, S5 Table).

**Fig 3:**
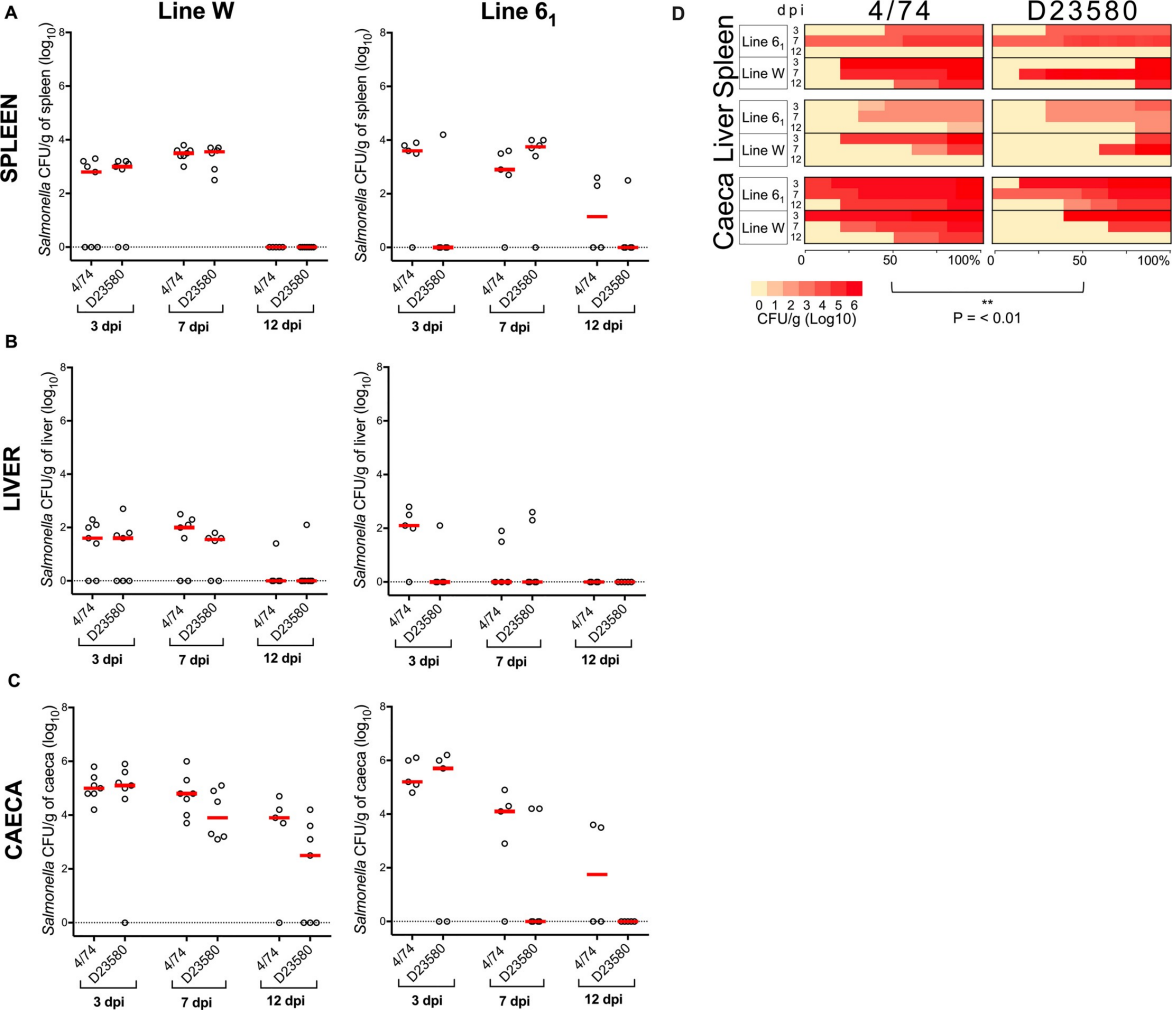
*S*. Typhimurium 4/74 is associated with an overall increased tissue burden in “*Salmonella-resistant”* inbred chickens relative to D23580. 14-day-old lines W and 6_1_ White Leghorn chickens were infected with 10^8^ CFU of *S*. Typhimurium 4/74 or D23580 and assessed at 3, 7 and 12 days post-oral infection. Each group contained four to seven birds. Statistical comparison was made using a Mann-Whitney test. NS: not significant, P > 0.05. Viable counts (CFU/g) of *S*. Typhimurium D23580 and 4/74 in spleen (A), liver (B) and caecal content (C) are shown. Counts are shown for individual birds with the bar representing the median value. A heatmap summarises the bacterial burden in all birds inoculated with 4/74 or D23580 (D). The heatmaps represents bacterial burden as a yellow (0 log_10_ CFU/g) to red (6 log_10_ CFU/g) colour scale and data are shown as percent of infected birds. Statistical modelling of the *“Salmonella-resistant”* inbred chicken infection data showed that 4/74 was associated with an average increased tissue burden of 0.6 log_10_ CFU/g tissue (P < 0.001).

Next, we compared the isolates in *“Salmonella-resistant”* inbred chickens from Line W and Line 6_1_. In these lines, differences between *S*. Typhimurium D23580 and 4/74 were more subtle, but showed a similar trend to the “*Salmonella-susceptible*” line data, with 4/74 causing a slightly higher tissue burden than D23580 (Fig 3). In general, the data show only small differences between colonisation levels of the 14-day-old *“Salmonella-susceptible”* and “*Salmonella-resistant*” chicken lines, consistent with reports that the *SAL1* resistance trait was only evident when one day-old chicks were challenged with *S*. Typhimurium [11].

Linear modelling of the *“Salmonella-resistant”* inbred chicken infection data showed that 4/74 was associated with an average increased tissue burden of 0.6 log_10_ CFU/g tissue, compared with infection by D23580 (P < 0.001, S6 Table).

When tissue burden data from 4/74 and D23580 in all five inbred lines were analysed with a linear model, strain 4/74 was associated with an overall average increased burden of 0.8 log_10_ CFU/g tissue relative to strain D23580 (P < 0.001, S4 Table). The model showed that the chicken line, tissue and timepoint were also significant variables that affected the tissue burden (S4 Table). Our findings show that statistical analyses involving multivariate comparison of the two bacterial strains across different chicken lines, tissue types and timepoints enabled the detection of subtle inter-strain variations in virulence.

### Infection by *S*. Typhimurium D23580 causes less pathology in inbred chicken lines than 4/74

**Fig 4:**
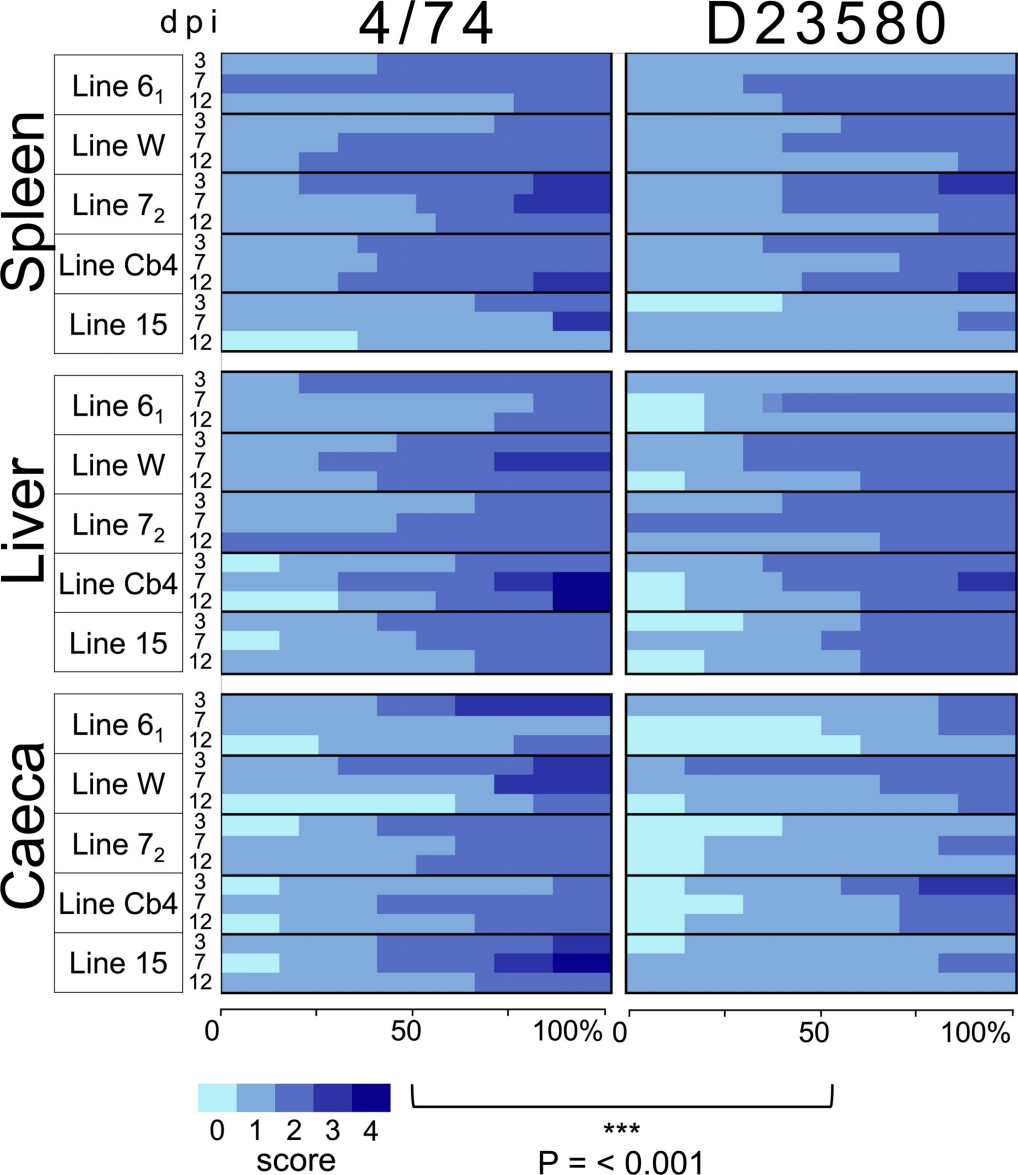
*S*. Typhimurium 4/74 caused more severe histopathological changes in spleen, liver and caeca of inbred chickens than D23580. Scores (0–4) based in histopathology score system used in Parsons *et al*, 2013 [3]. The histopathological tissue changes were semi-quantitatively measured using a grading scale ranging from 0 (no change) to 4 (severe damage to the tissue). Data is represented as a heatmap showing the histopathology score as a colour gradient by percent of birds. Statistical modelling showed that strain 4/74 was associated with an average increase of 0.22 relative to D23580 in the tissue histopathology scoring system (P < 0.001, S9–S11 Tables).

Severity of infection may not necessarily correlate with bacterial infection burden, for example if one strain possesses enhanced virulence factors, a lower bacterial burden could cause the same severity of disease. Therefore, to qualitatively compare the infection biology of D23580 and 4/74, we assessed histopathological changes associated with the infection by these two isolates in caeca, liver and spleen of infected birds at 3, 7 and 12 dpi. Similar tissue changes were observed in the five inbred chicken lines; there was no difference in the nature of the histopathological changes caused by bacterial strain D23580 or 4/74 in the different chicken lines. However, tissue changes did vary in severity with 4/74 causing more pronounced pathology than D23580 (Fig 4). Changes in the caecum include variable degrees of heterophilic inflammatory infiltrates within the lamina propia, extending to intra-epithelial populations. Lesions in the liver varied from small clusters of often perivascular macrophages or heterophils, to multifocally scattered larger accumulations, sometimes exhibiting the morphology of small dense aggregates of macrophages, reminiscent of a granuloma. Meanwhile, splenic lesions consisted of increased numbers of inflammatory cells, particularly macrophages, occupying the red pulp and in more severe cases, also including the white pulp.

The data indicated that both isolates D23580 and 4/74 caused similar tissue changes during chicken infection, though 4/74 was associated with consistently higher histopathological scores than D23580 (as illustrated by the dark blue-shift in the heatmaps shown in Fig 4, and data in S7 and S8 Tables). The observed pathological changes were consistent with the bacterial colonisation levels present in the tissues of individual infected birds, with a higher pathology score being associated with a greater bacterial load. Statistical modelling showed that strain 4/74 was associated with an average increase of 0.22 in the tissue histopathology scoring system relative to D23580 (P < 0.001, S9–S11 Tables), supporting the trend shown in Fig 4.

Overall, the colonisation levels and histopathological analysis of infected inbred and outbred chickens in this study differed from previous findings with outbred birds [3]. The key difference between the two studies is that here we infected a total of 113 birds per strain representing one outbred line and five inbred lines, whereas Parsons *et al*. (2013) [3] used 20 outbred birds per strain. Here we did not observe the statistically-significant difference that had been reported previously in outbred birds [3], where ST313 strain D23580 was associated with increased virulence relative to ST19 strain 4/74. Further, when genetically homogeneous inbred chickens were used, ST313 strain D23580 showed a reduced virulence phenotype relative to ST19 strain 4/74, in terms of both tissue burden and pathology.

Our data highlight the challenge of achieving reproducibility in animal experiments, and the importance of sample size in infection research. We conclude that there are limitations to the use of animal models when investigating differences in virulence between closely-related strains. Additionally, we note that the small but consistent inter-strain differences detected here were clear only when utilising linear modelling analysis, which allowed global comparison of strains across different tissue types and timepoints. Simple pairwise comparisons between strains in specific tissue types and time points rarely detected differences between the strains (Fig 2).

### Differences in the virulence of *S*. Typhimurium 4/74 and D23580 in alternative infection models

**Fig 5:**
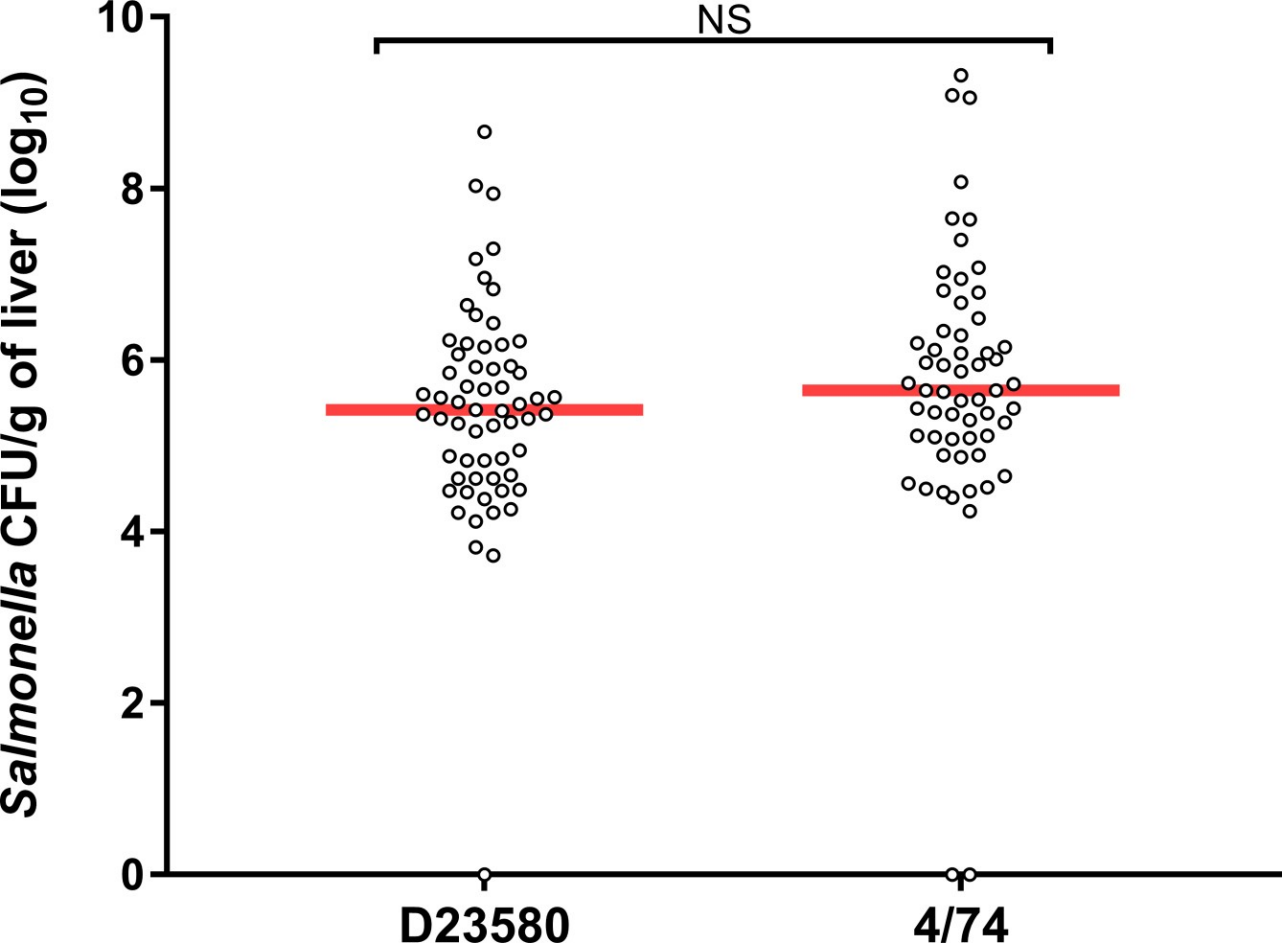
Similar bacterial loads in the liver of chick embryos inoculated with *S*. Typhimurium D23580 and 4/74. Viable counts (CFU/g) of *S*. Typhimurium 4/74 and D23580 in liver at 16 hours post-infection with 10^2^ CFU bacteria. Counts are shown for individual embryos with the bar representing the median value. Each isolate was tested in sixty embryos. Statistical comparison was made using a t-test. NS: not significant, P > 0.05.

To build on our findings, we studied 4/74 and D23580 in other infection models. For this, we used alternative infection models that did not require a UK Home Office licence or ethical approval. The first of these was the chick embryo infection model [16]. The embryos used in this model are classified as non-protected under the UK Animals (Scientific Procedures) Act 1986 since the experiments are carried out at embryonic day 11 and the embryos were euthanised before embryonic day 14; only animals at embryonic age 14 or older are covered by the act [26]. The chick embryo infection model involves the injection of bacteria into the allantoic cavity of the egg, in this model only virulent bacteria are able to migrate from this site to reach other compartments of the egg, including the embryo and yolk. Meanwhile, attenuated bacteria remain confined to the allantoic cavity [16]. Virulence is assessed by determining the colonisation levels in the embryo liver after a specific incubation period. We used an incubation period of 16 hours post-inoculation. Longer incubation times led to mortality, rapid tissue destruction, and necrosis which prevented meaningful measurements (S1 Data set). At 16 hours post-inoculation, similar levels of colonisation by D23580 and 4/74 were found in the embryos, around 10^5^ CFU/g of liver (Fig 5).

**Fig 6:**
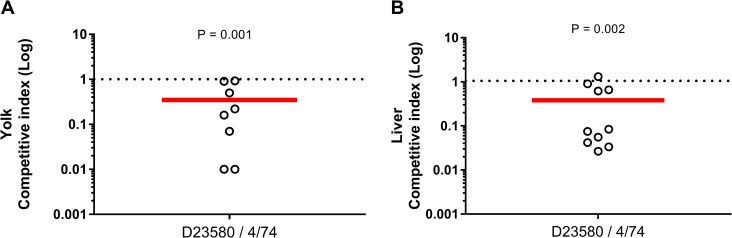
D23580 is less fit than 4/74 in the chick embryo model. *S*. Typhimurium 4/74 and D23580 at 16 hours post-infection with 10^2^ CFU bacteria. Competitive index in yolk (A) and liver (B). Competitive index values were calculated as the ratio of D23580 to 4/74 recovered from the yolk or liver, divided by the ratio of D23580 to 4/74 present in the inoculum. The numbers of each strain were quantified by plating dilution series on LB agar using antibiotic resistance to distinguish between strains. Counts are shown as individual embryos with the bar representing the median value. Ten and eight embryos were used to measure the competitive index in yolk and liver respectively. Statistical comparison was made using a t-test and shows that CI values were statistically different from 1.0.

To confirm the embryo liver-colonisation level data, we performed a competitive index assay by simultaneously inoculating embryonated eggs with equal quantities of both isolates. At 16 hours post-inoculation, in both liver and yolk, the D23580 isolate exhibited CI values < 1.0, indicating that fitness of D23580 was reduced compared to 4/74 at a statistically significant level (Fig 6). The CI of D23580 vs 4/74 was 0.38 (P = 0.001) in egg yolk and 0.34 (P = 0.002) in liver.

**Fig 7:**
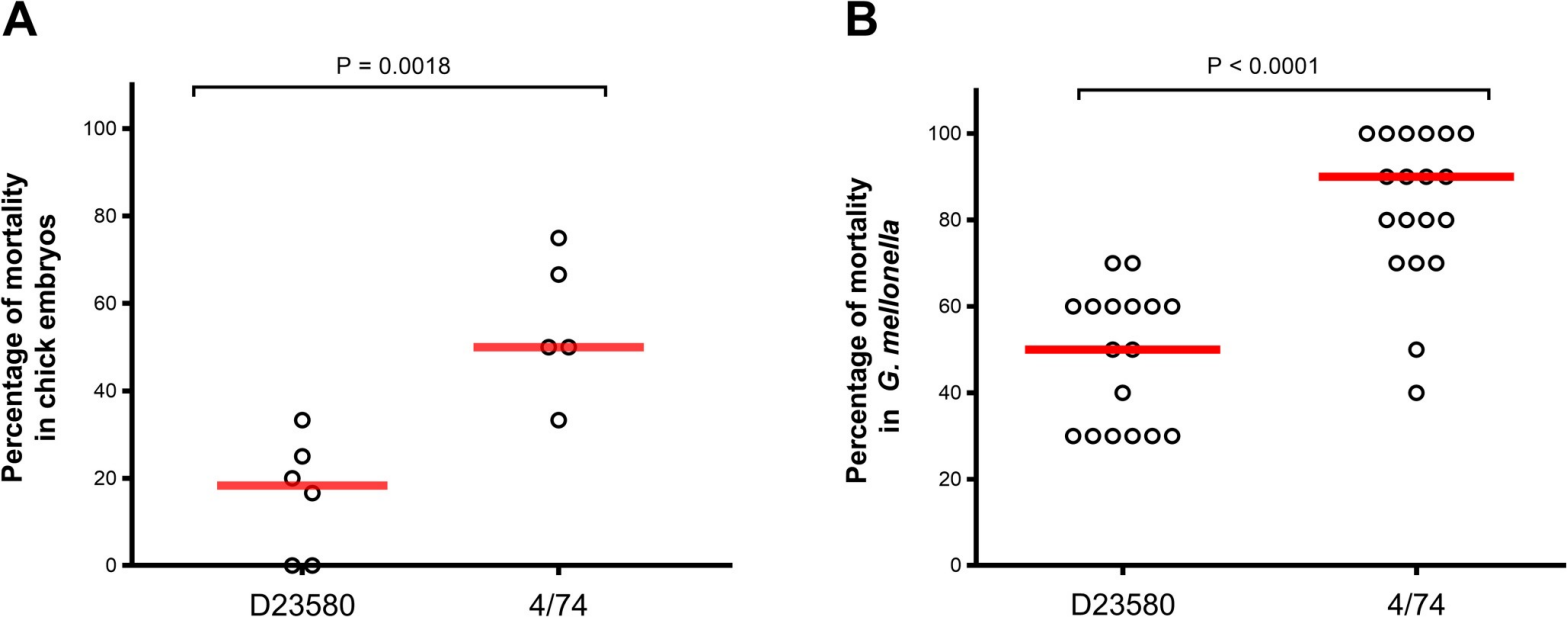
*S*. Typhimurium 4/74 causes greater mortality than D23580 in two alternative infection models. Mortality rate in chick embryos (A) and *G*. *mellonella* larvae (B) inoculated with *S*. Typhimurium 4/74 and D23580 for both infection models. Data are represented as percentage mortality at 24 hours post-infection with 10^2^ CFU bacteria. Bar represents the median value.

Next, we assessed the ability of the bacteria to kill chick embryos, and found that isolate 4/74 caused higher mortality than D23580 at 24 hours post inoculation (P = 0.0018; Fig 7A).

As *Salmonella* infection of chick embryos is not an established model, we tested the ability of a number of mutants that lacked important *Salmonella* virulence factors to infect the embryos. Our data showed that mutants deficient in LPS and flagella biosynthesis were attenuated in the chick embryo model, but SPI-1, SPI-2 and the PhoPQ regulon were not required for infection (S12 Table and S2 Fig); further investigation is needed to confirm this finding.

Lastly, we compared the levels of mortality induced by D23580 and 4/74 in the wax moth *G*. *mellonella* larvae model [17]. The lymph of *G*. *mellonella* larvae contains haemocytes (phagocytic cells that share many properties with mammalian phagocytes), representing a good model for the innate immunity response [27]. The *G*. *mellonella* larvae were infected by inoculation of bacteria via injection into the haemocoel, and percentage mortality caused by each of the two isolates was assessed at 24 hours post-inoculation. The 4/74 isolate caused a higher mortality rate than D23580 (P<0.0001; Fig 7B), consistent with our finding that 4/74 was more fit than D23580 in the chick embryo model.

## Discussion

Our work highlights the importance of sample size, choice of host and sampling protocol in animal infection research. Previous findings that *S*. Typhimurium ST313 strain D23580 had a more invasive phenotype than ST19 strain 4/74 in the chicken model [3] were not reproduced in either outbred or inbred chicken lines in this study.

In their review, Casadevall and Fang [28] suggested that sample size in infection research is a crucial variable, and that the use of too few animals can obscure significant differences that would be evident in larger populations. Although increasing the number of animals reduces the probability of false negative or false positive results, experimentation with vertebrate animals is limited by the Principles of the 3Rs that advocate the reduction of the number of animals used in experiments [26,29]. An unintended consequence of the Principles of the 3R’s is that different laboratories rarely repeat experiments to verify key virulence differences that have been reported to distinguish bacterial isolates, or that differentiate wild-type from mutant bacteria. We note that experimental chicken infections have been reported to have their own limitations, even when genetically homogeneous inbred lines are used [14]. Variations in the levels of colonisation of infected chickens could reflect stochasticity in oral infections, natural differences in the microbiota, maternally-derived immunity or simple variation in size and fitness of the experimental animals.

The use of alternative infection models such as the chicken embryo or the wax moth *G*. *mellonella* larvae model allow large sample sizes without compromising ethical standards, whilst maintaining genetic homogeneity. Through the use of multiple alternative infection models and large sample sizes, we detected consistent significant differences between ST313 and ST19 strains of *S*. Typhimurium, suggesting alternative infection models represent promising alternatives to vertebrate infection models where differences between strains in question may be nuanced.

Overall, our data suggest that the qualitative nature of disease caused by *S*. Typhimurium ST313 and ST19 isolates (D23580 and 4/74) in avian and insect models is broadly similar. However, ST313 shows reduced levels of colonisation of inbred chickens, relative to ST19. The use of alternative infection models showed that *S*. Typhimurium ST313 exhibited reduced virulence in chick embryos and *G*. *mellonella* larvae. It remains likely that African *S*. Typhimurium ST313 has a host-specific adaptation that is responsible for systemic infection in humans but was not apparent in the animal models of infection tested here.

Human tissue culture models of infection have failed to identify consistent differences in infection biology between ST313 and ST19 strains [10]. Furthermore, the variety of animal models that have been used to study African *Salmonella* and to compare ST313 with ST19 did not find a consistent ST313-associated phenotype. The animal infection data presented here do confirm that ST313 does not share the host-restricted lifestyle of *S*. Typhi, *S*. Gallinarum or *S*. Abortusequi, but the chicken and insect-based models only revealed subtle phenotypic differences between ST313 from ST19.

One model, the streptomycin-treated mouse, has been used effectively to demonstrate the importance of pseudogenisation of the *sseI* effector gene of S. Typhimurium ST313 for dendritic cell-mediated dissemination [30]. One explanation for the efficacy of this murine model is that the effect of streptomycin treatment in some ways mimics the immune-compromised human population to which *S*. Typhimurium ST313 is niche-adapted. It is possible that the clinical usage of broad-spectrum antibiotics amongst the immunocompromised *S*. Typhimurium ST313-susceptible African populations is a significant factor. The host immune-deficiency, which occurs in African populations suffering from HIV, malaria and malnutrition, or an antibiotic-induced gut microbiota disturbance, may be critical for studying the infection biology of ST313 strains. Immune-deficient infection models are in development, including HIV-infected macrophages [31], and malaria-infected mice [32], and could prove critical for understanding the genetic mechanism of adaption of African *S*. Typhimurium ST313 to an invasive lifestyle.

## Supporting information

S1 Table Bacterial strains. (DOCX)

S2 Table Oligonucleotides used to construct knock-out mutants used in this study. (DOCX)

S3 Table Summary of linear model predicting infection burden (log_10_CFU/g tissue) of outbred chickens as a function of strain, tissue and timepoint. (DOCX)

S4 Table Summary of linear model predicting infection burden (log_10_CFU/g tissue) as a function of timepoint, chicken line, strain and tissue, including data from both “*Salmonella-resistant”* and *Salmonella-susceptible”* chicken lines. (DOCX)

S5 Table Summary of linear model predicting infection burden (log_10_CFU/g tissue) as a function of timepoint, chicken line, strain and tissue for *“Salmonella-susceptible”* lines only. (DOCX)

S6 Table Summary of linear model predicting infection burden (log_10_CFU/g tissue) as a function of timepoint, chicken line, strain and tissue for “*Salmonella-resistant”* lines only. (DOCX)

S7 Table Histopathology scores in spleen, liver and caeca of inbred chickens infected with *S*. Typhimurium D23580 or 4/74. Scores based in histopathology score system used in Parsons et al, 2013 [3]. Mean, median and range are presented.
(DOCX)

S8 Table Description of histopathological changes in spleen, liver and caeca of inbred chickens infected with *S*. Typhimurium D23580 or 4/74. The qualitative assessment of the histopathological changes associated with infection at 3, 7 and 12 dpi was made by a veterinary pathologist.
(DOCX)

S9 Table Summary of linear model predicting histopathological scores as a function of timepoint, chicken line, strain and tissue, including data from both resistant and susceptible chicken lines. (DOCX)

S10 Table Summary of linear model predicting histopathological scores as a function of timepoint, chicken line, strain and tissue, including data from “*Salmonella-susceptible”* lines only. (DOCX)

S11 Table Summary of linear model predicting histopathological scores as a function of timepoint, chicken line, strain and tissue, including data from “*Salmonella-resistant”* lines only. (DOCX)

S12 Table Virulence of attenuated *S*. Typhimurium strains assessed in the chick embryo model. (DOCX)

S1 Fig **Viable counts (CFU/g) of *S*. Typhimurium 4/74 and D23580 in spleen (A), liver (B) and caecal content (C) of outbred chickens at 1 and 5 dpi.** 14-day-old Lohman Brown Layers inoculated with 10^8^ CFU. Counts are shown as individual birds with the bar representing the median value. Eight birds per group. Statistical comparison was made using a Mann-Whitney test. NS: not significant, P > 0.05.
(TIF)

S2 Fig Bacterial counts in the liver after infection of chicken embryos. Attenuated mutants in the 4/74 (A) and D23580 (B) genetic background were tested. Bars represent the median values; circles represent individual embryos. Data from three independent experiments. Six embryos per group were used in each replicate. Individual groups (wild-type versus mutant) were compared using Mann-Whitney U test.
(TIF)

S1 Dataset Raw data for Figs 1–7. (XLS)

S1 Text Salmonella virulence in the chick embryo model. (DOCX)
