# Supplementary material for: The use of chicken and insect infection models to assess the virulence of African Salmonella Typhimurium ST313
Source: PLoS Negl Trop Dis. 2019 Jul 26;13(7):e0007540. doi: 10.1371/journal.pntd.0007540 (PMC6685681; doi:10.1371/journal.pntd.0007540)
Supplement: S1 Table — (DOCX) [file pntd.0007540.s001.docx]

| **Bacterial strains** | **Description** | **Reference/origin** |
| --- | --- | --- |
| ***S.* Typhimurium D23580 derivatives** |  |  |
| JH3621 | D23580, wild-type | Kingsley et al., 2009 [1] |
| JH4328 | Δ*hilC::frt* | This study |
| SO-53 | Δ*ssrAB::frt* | This study |
| JH4187 | Δ*waaG::frt* | This study (*aph* gene removed by FLP recombinase [2] |
| SO-51 | Δ*waaL::frt* | This study |
| JH3950 | IGR *STMMW_41451-41461*::*tetRA* | This study |
| JH4235 | Δ*rpoE::frt* | This study |
| ***S.* Typhimurium 4/74 derivatives** |  |  |
| JH3291 | 4/74, wild-type | Rankin and Taylor, 1966 [3] |
| JH3635 | Δ*hilD::frt* | P22 transduction from JVS-5924 |
| JH4280 | Δ*invA::frt* | This study |
| JH3660 | Δ*phoPQ::frt* | Colgan et al., 2016 [4] |
| JH4188 | Δ*waaG::frt* | This study (*aph* gene removed by FLP recombinase [2] |
| JH4186 | Δ*fljAB::frt* Δ*fliC::frt* | This study |
| JH4279 | Δ*motA::frt* | This study |
| JH4284 | Δ*SL1483::aph* | This study |
| JH3630 | Δ*rpoE::frt* | P22 transduction from JVS-1028 |

**References**

1. Kingsley RA, Msefula CL, Thomson NR, Kariuki S, Holt KE, Gordon MA, et al. Epidemic multiple drug resistant Salmonella Typhimurium causing invasive disease in sub-Saharan Africa have a distinct genotype. Genome Res. 2009;19: 2279–2287. doi:10.1101/gr.091017.109

2. Hammarlöf DL, Kröger C, Owen SV, Canals R, Lacharme-Lora L, Wenner N, et al. Role of a single noncoding nucleotide in the evolution of an epidemic African clade of Salmonella. Proc Natl Acad Sci U S A. 2018;115: E2614–E2623. doi:10.1073/pnas.1714718115

3. Rankin JD, Taylor RJ. The estimation of doses of Salmonella typhimurium suitable for the experimental production of disease in calves. Vet Rec. 1966;78: 706–707.

4. Colgan AM, Kröger C, Diard M, Hardt W-D, Puente JL, Sivasankaran SK, et al. The Impact of 18 Ancestral and Horizontally-Acquired Regulatory Proteins upon the Transcriptome and sRNA Landscape of Salmonella enterica serovar Typhimurium. Casadesús J, editor. PLOS Genetics. 2016;12: e1006258. doi:10.1371/journal.pgen.1006258
