# Supplementary material for: The use of chicken and insect infection models to assess the virulence of African Salmonella Typhimurium ST313
Source: PLoS Negl Trop Dis. 2019 Jul 26;13(7):e0007540. doi: 10.1371/journal.pntd.0007540 (PMC6685681; doi:10.1371/journal.pntd.0007540)
Supplement: S2 Table — (DOCX) [file pntd.0007540.s002.docx]

| **Oligonucleotide name** | **Sequence 5’→3’** | **Oligo purpose** |
| --- | --- | --- |
| Fw-hilC-P1 | CACACAGGATAAAATATGGTATTGCCTTCAATGAATAAATCAGTTGTGTAGGCTGGAGCTGCTTC | Construction of strain JH3635 |
| Rv-hilC-P2 | AAATAATTTCACAAATCAATGGTTCATTGTACGCATAAAGCTAAGCATATGAATATCCTCCTTAG |  |
| Fw-rpoE-P1 | TGGTTTGGGGAGACATTACCTCGGATGAGCGAGCAGTTAACGGACGTGTAGGCTGGAGCTGCTTC | Construction of strain JH3630 |
| Rv-rpoE-P2 | ACCTTTTCCAGTATCCCGCTATCGTCAACGCCTGATAAGCGGTTGCATATGAATATCCTCCTTAG |  |
| Fw-invA-P1 | ACTATTGAAAAGCTGTCTTAATTTAATATTAACAGGATACCTATAGTGTAGGCTGGAGCTGCTTC | Construction of strain JH4280 |
| Rv-invA-P2 | CAGCGATATCCAAATGTTGCATAGATCTTTTCCTTAATTAAGCCCCATATGAATATCCTCCTTAG |  |
| Fw-fljAB-P1 | TCATGGAGGATTGCTTTATCAAAAACCTTCCAAAAGGAAAATTTTGTGTAGGCTGGAGCTGCTTC | Construction of strain JH4186 |
| Rv-fljAB-P2 | CCCTAAGTTTTACTTTTCTCACGGAATTTTTTATTACCGTAGGCGCATATGAATATCCTCCTTAG |  |
| Fw-fliC-P1 | GCAACAGCCCAATAACATCAAGTTGTAATTGATAAGGAAAAGATCGTGTAGGCTGGAGCTGCTTC | Construction of strain JH4186 |
| Rv-fliC-P2 | CGCTGCCTTGATTGTGTACCACGTGTCGGTGAATCAATCGCCGGACATATGAATATCCTCCTTAG |  |
| Fw-motA-P1 | TTAGGTTACCTGGTGGTTATCGGTACAGTTTTCGGCGGTTATGTCGTGTAGGCTGGAGCTGCTTC | Construction of strain JH4279 |
| Rv-motA-P2 | GTTTTACGACGACAATGGGATGAGCCTGATTTTTCATGCTTCCTCCATATGAATATCCTCCTTAG |  |
| ins_STnc230_rev | CCGGGAGCACTACCGTGATTTTGGCCCCACGCTGGCGCTGGAAAATCTGGGTCCATATGAATATCCTCCTTAG | Construction of strain JH4284 |
| del_SL1483_for | TTCACAAACAGCAGGTGCATCAGGCGACTGGTGGCATAGTCAACATAGACGTGTAGGCTGGAGCTGCTTC |  |
| NW_202 | CATTCAAGATGTGAATGTAATTTATTTGTTTATAATGTTATTAATATATGTTAAGACCCACTTTCACATT | Construction of strain JH3950 |
| NW_203 | GAAAATATAAAAGACAAAACAATCATTAAAACATTTATCAACTTACATTACTAAGCACTTGTCTCCTG |  |
| ssrA_KO_F | TAAGGCTCATCAAAATATGACCAATGCTTAATACCATCGGACGCCCCTGGGTGTAGGCTGGAGCTGCTTC | Construction of strain SO-53 |
| ssrB_KO_R | TAGTGATCAAGTGCCAAAGATTTTGCAACAGGCAACTGGAGGGAAGCATTCATATGAATATCCTCCTTA |  |
| waaL_del_fw | agattcattaaagagactctgtctcatcccaaacctattgtggagaaaagGTGTAGGCTGGAGCTGCTTC | Construction of strain SO-51 |
| waaL_del_rv | cctgatgatggaaaacgcgctgataccgtaataagtatcagcgcgtttttCATATGAATATCCTCCTTAG |  |
