# Supplementary material for: The use of chicken and insect infection models to assess the virulence of African Salmonella Typhimurium ST313
Source: PLoS Negl Trop Dis. 2019 Jul 26;13(7):e0007540. doi: 10.1371/journal.pntd.0007540 (PMC6685681; doi:10.1371/journal.pntd.0007540)
Supplement: S3 Table — (DOCX) [file pntd.0007540.s003.docx]

| Residuals:  Min 1Q Median 3Q Max  -4.5361 -0.7061 -0.1204 0.8347 3.6713 | | | | | | | | | |
| --- | --- | --- | --- | --- | --- | --- | --- | --- | --- |
| Coefficients: | | | | | | | | | |
|  | Estimate | | Std. Error | | t value | | Pr(>\|t\|) | |  |
| (Intercept) | 3.9733 | | 0.2792 | | 14.229 | | <2e-16 | | *** |
| Strain = D23580 | 0.1269 | | 0.2231 | | 0.569 | | 0.5706 | |  |
| Tissue = liver | -3.9797 | | 0.2731 | | -14.572 | | <2e-16 | | *** |
| Tissue = spleen | -3.2893 | | 0.2731 | | -12.044 | | <2e-16 | | *** |
| Timepoint = 3 dpi | 0.436 | | 0.2748 | | 1.586 | | 0.115 | |  |
| Timepoint = 5 dpi | 0.5045 | | 0.2748 | | 1.836 | | 0.0686 | | . |
|  | | | | | | | | | |
| Residual standard error: 1.324 on 135 degrees of freedom  Multiple R-squared: 0.6463, Adjusted R-squared: 0.6332  F-statistic: 49.34 on 5 and 135 DF, p-value: < 2.2e-16 | | | | | | | | | |
|  | | | | | | | | | |
| Response: *Salmonella* CFU/g tissue (log_10_) | | | | | | | | | |
|  | Sum Sq | Df | | F value | | Pr(>F) | |  | |
| (Intercept) | 354.86 | 1 | | 202.4617 | | <2e-16 | | *** | |
| Strain | 0.57 | 1 | | 0.3232 | | 0.5706 | |  | |
| Tissue | 425.1 | 2 | | 121.2693 | | <2e-16 | | *** | |
| Timepoint | 6.88 | 2 | | 1.9628 | | 0.1445 | |  | |
| Residuals | 236.62 | 135 | |  | |  | |  | |

Significance levels: ‘***’ =0.001; ‘**’ =0.01, ‘*’ =0.05; ‘.’ =0.1; ‘ ’ =1
