# Supplementary material for: The use of chicken and insect infection models to assess the virulence of African Salmonella Typhimurium ST313
Source: PLoS Negl Trop Dis. 2019 Jul 26;13(7):e0007540. doi: 10.1371/journal.pntd.0007540 (PMC6685681; doi:10.1371/journal.pntd.0007540)
Supplement: S4 Table — (DOCX) [file pntd.0007540.s004.docx]

| Residuals:  Min 1Q Median 3Q Max  -3.5266 -1.1826 0.2128 1.2179 4.0004 | | | | | | | | | |
| --- | --- | --- | --- | --- | --- | --- | --- | --- | --- |
| Coefficients: | | | | | | | | | |
|  | Estimate | | Std. Error | | t value | | Pr(>\|t\|) | |  |
| (Intercept) | 2.96E+00 | | 2.09E-01 | | 1.42E+01 | | <2.00E-16 | | *** |
| Line = 6 vs 15 | 3.53E-02 | | 2.22E-01 | | 1.59E-01 | | 8.74E-01 | |  |
| Line = 7 vs 15 | 4.00E-01 | | 2.24E-01 | | 1.78E+00 | | 7.52E-02 | | . |
| Line = Cb4 vs 15 | 1.53E-01 | | 2.05E-01 | | 7.48E-01 | | 4.55E-01 | |  |
| Line = W vs 15 | 5.65E-01 | | 2.07E-01 | | 2.73E+00 | | 6.62E-03 | | ** |
| Strain = D23580 vs 4/74 | -8.06E-01 | | 1.37E-01 | | -5.87E+00 | | 7.84E-09 | | *** |
| Tissue = liver vs caeca | -1.61E+00 | | 1.68E-01 | | -9.59E+00 | | <2.00E-16 | | *** |
| Tissue = spleen vs caeca | -2.15E-01 | | 1.68E-01 | | -1.28E+00 | | 2.00E-01 | |  |
| Timepoint = 7 dpi vs 3 dpi | 1.64E-01 | | 1.65E-01 | | 9.96E-01 | | 3.20E-01 | |  |
| Timepoint = 12 dpi vs 3 dpi | -1.01E+00 | | 1.70E-01 | | -5.95E+00 | | 4.89E-09 | | *** |
|  | | | | | | | | | |
| Residual standard error: 1.584 on 524 degrees of freedom  Multiple R-squared: 0.288, Adjusted R-squared: 0.2757  F-statistic: 23.55 on 9 and 524 DF, p-value: < 2.2e-16 | | | | | | | | | |
|  | | | | | | | | | |
| Response: *Salmonella* CFU/g tissue (log_10_) | | | | | | | | | |
|  | Sum Sq | Df | | F value | | Pr(>F) | |  | |
| (Intercept) | 494.17 | 1 | | 2.04E+02 | | <2.00E-16 | | *** | |
| Line | 25.95 | 4 | | 2.67E+00 | | 3.13E-02 | | * | |
| Strain | 86.42 | 1 | | 3.56E+01 | | 4.44E-09 | | *** | |
| Tissue | 146.46 | 2 | | 3.02E+01 | | 3.97E-13 | | *** | |
| Timepoint | 102.22 | 2 | | 2.11E+01 | | 1.60E-09 | | *** | |
| Tissue * Timepoint | 53.6 | 4 | | 5.52E+00 | | 2.32E-04 | | *** | |
| Residuals | 1261.7 | 520 | |  | |  | |  | |

Significance levels: ‘***’ =0.001; ‘**’ =0.01, ‘*’ =0.05; ‘.’ =0.1; ‘ ’ =1
