# Supplementary material for: The use of chicken and insect infection models to assess the virulence of African Salmonella Typhimurium ST313
Source: PLoS Negl Trop Dis. 2019 Jul 26;13(7):e0007540. doi: 10.1371/journal.pntd.0007540 (PMC6685681; doi:10.1371/journal.pntd.0007540)
Supplement: S5 Table — (DOCX) [file pntd.0007540.s005.docx]

| Residuals:  Min 1Q Median 3Q Max   \| -3.627 -1.104 0.137 1.006. 3.967 \| \| --- \| | | | | | | | | | |
| --- | --- | --- | --- | --- | --- | --- | --- | --- | --- | --- |
| Coefficients: | | | | | | | | | |
|  | Estimate | | Std. Error | | t value | | Pr(>\|t\|) | |  |
| (Intercept) | 2.3691 | | 0.235 | | 10.08 | | <2.00E-16 | | *** |
| Line = 7 | 0.4002 | | 0.2192 | | 1.826 | | 0.06881 | | . |
| Line = Cb4 | 0.1501 | | 0.2 | | 0.751 | | 0.45349 | |  |
| Strain = D23580 | -0.9028 | | 0.1714 | | -5.268 | | 2.54E-07 | | *** |
| Tissue = liver | -1.0396 | | 0.2097 | | -4.958 | | 1.16E-06 | | *** |
| Tissue = spleen | 0.5725 | | 0.2097 | | 2.731 | | 0.00667 | | ** |
| Timepoint = 7 dpi | 0.2856 | | 0.2064 | | 1.384 | | 0.16743 | |  |
| Timepoint = 12 dpi | -0.5309 | | 0.211 | | -2.515 | | 0.01238 | | * |
|  | | | | | | | | | |
| Residual standard error: 1.584 on 319 degrees of freedom  Multiple R-squared: 0.253, Adjusted R-squared: 0.237  F-statistic: 15.44 on 7 and 319 DF, p-value: < 2.20e-16 | | | | | | | | | |
|  | | | | | | | | | |
| Response: *Salmonella* CFU/g tissue (log_10_) | | | | | | | | | |
|  | Sum Sq | Df | | F value | | Pr(>F) | |  | |
| (Intercept) | 217.9 | 1 | | 92.237 | | <2.00E-16 | | *** | |
| Line | 8.02 | 2 | | 1.6976 | | 0.1847914 | |  | |
| Strain | 66.5 | 1 | | 28.1495 | | 2.12E-07 | | *** | |
| Tissue | 40.1 | 2 | | 8.4865 | | 0.0002572 | | *** | |
| Timepoint | 30.28 | 2 | | 6.4099 | | 0.001868 | | ** | |
| Tissue * Timepoint | 20.19 | 4 | | 2.137 | | 0.076087 | | . | |
| Residuals | 744.14 | 315 | |  | |  | |  | |

Significance levels: ‘***’ =0.001; ‘**’ =0.01, ‘*’ =0.05; ‘.’ =0.1; ‘ ’ =1
