# Supplementary material for: The use of chicken and insect infection models to assess the virulence of African Salmonella Typhimurium ST313
Source: PLoS Negl Trop Dis. 2019 Jul 26;13(7):e0007540. doi: 10.1371/journal.pntd.0007540 (PMC6685681; doi:10.1371/journal.pntd.0007540)
Supplement: S6 Table — (DOCX) [file pntd.0007540.s006.docx]

| Residuals:  Min 1Q Median 3Q Max   \| -3.8914 -0.7819 0.3475 1.0309. 2.8984 \| \| --- \| | | | | | | | | | |
| --- | --- | --- | --- | --- | --- | --- | --- | --- | --- | --- |
| Coefficients: | | | | | | | | | |
|  | Estimate | | Std. Error | | t value | | Pr(>\|t\|) | |  |
| (Intercept) | 3.92401 | | 0.27308 | | 14.369 | | <2.00E-16 | | *** |
| Line = W | 0.5325 | | 0.20381 | | 2.613 | | 0.00966 | | ** |
| Strain = D23580 | -0.63041 | | 0.20256 | | -3.112 | | 0.00213 | | ** |
| Tissue = liver | -2.51166 | | 0.24726 | | -10.158 | | <2.00E-16 | | *** |
| Tissue = spleen | -1.45981 | | 0.24726 | | -5.904 | | 1.50E-08 | | *** |
| Timepoint = 7 dpi | -0.03257 | | 0.24221 | | -0.134 | | 0.89316 | |  |
| Timepoint = 12 dpi | -1.78413 | | 0.25098 | | -7.109 | | 2.03E-11 | | *** |
|  |  | |  | |  | |  | |  |
| Residual standard error: 1.452 on 200 degrees of freedom  Multiple R-squared: 0.4858, Adjusted R-squared: 0.4703  F-statistic: 31.49 on 6 and 200 DF, p-value: < 2.20e-16 | | | | | | | | | |
|  | | | | | | | | | |
| Response: *Salmonella* CFU/g tissue (log_10_) | | | | | | | | | |
|  | Sum Sq | Df | | F value | | Pr(>F) | |  | |
| (Intercept) | 405.03 | 1 | | 214.4893 | | <2.00E-16 | | *** | |
| Line | 14.4 | 1 | | 7.6256 | | 0.006301 | | ** | |
| Strain | 20.43 | 1 | | 10.819 | | 0.001191 | | ** | |
| Tissue | 158.73 | 2 | | 42.0303 | | 6.46E-16 | | *** | |
| Timepoint | 90.09 | 2 | | 23.8546 | | 5.34E-10 | | *** | |
| Tissue * Timepoint | 51.75 | 4 | | 6.8509 | | 3.51E-05 | | *** | |
| Residuals | 370.11 | 196 | |  | |  | |  | |

Significance levels: ‘***’ =0.001; ‘**’ =0.01, ‘*’ =0.05; ‘.’ =0.1; ‘ ’ =1
