# Supplementary material for: The use of chicken and insect infection models to assess the virulence of African Salmonella Typhimurium ST313
Source: PLoS Negl Trop Dis. 2019 Jul 26;13(7):e0007540. doi: 10.1371/journal.pntd.0007540 (PMC6685681; doi:10.1371/journal.pntd.0007540)
Supplement: S7 Table — Scores based in histopathology score system used in Parsons et al, 2013 [3]. Mean, median and range are presented. (DOCX) [file pntd.0007540.s007.docx]

| **Line 6_1_** | | | | | | | | | | | | |
| --- | --- | --- | --- | --- | --- | --- | --- | --- | --- | --- | --- | --- |
| **Tissue** | **3 dpi** | | | | **7 dpi** | | | | **12 dpi** | | | |
|  | **D23580** | | **4/74** | | **D23580** | | **4/74** | | **D23580** | | **4/74** | |
|  | Mean | Median (range) | Mean | Median (range) | Mean | Median (range) | Mean | Median (range) | Mean | Median (range) | Mean | Median (range) |
| **Caecum** | 1.2 | 1 (1-2) | 2 | 2 (1-3) | 0.7 | 0.5 (0-2) | 1 | 1 (1-1) | 0.4 | 0 (0-1) | 1 | 1.5 (0-2) |
| **Liver** | 1 | 1 (1-1) | 1.8 | 2 (1-2) | 1.5 | 2 (0-2) | 1.2 | 1 (1-2) | 0.8 | 1 (0-1) | 1.2 | 1 (1-2) |
| **Spleen** | 1 | 1 (1-1) | 1.6 | 2 (1-2) | 1.7 | 2 (1-2) | 2 | 2 | 1.6 | 2 (1-2) | 1.2 | 1 (1-2) |
| **Line W** | | | | | | | | | | | | |
| **Tissue** | **3 dpi** | | | | **7 dpi** | | | | **12 dpi** | | | |
|  | **D23580** | | **4/74** | | **D23580** | | **4/74** | | **D23580** | | **4/74** | |
|  | Mean | Median (range) | Mean | Median (range) | Mean | Median (range) | Mean | Median (range) | Mean | Median (range) | Mean | Median (range) |
| **Caecum** | 1.9 | 2 (1-2) | 1.7 | 2 (1-3) | 1.3 | 1 (1-2) | 1.4 | 1 (1-3) | 1 | 1 (0-2) | 0.6 | 0 (0-2) |
| **Liver** | 1.7 | 2 (1-2) | 1.5 | 2 (1-2) | 1.7 | 2 (1-2) | 2 | 2 (1-3) | 1.3 | 1 (0-2) | 1.6 | 2 (1-2) |
| **Spleen** | 1.4 | 1 (1-2) | 1.3 | 1 (1-2) | 1.6 | 2 (1-2) | 1.7 | 2 (1-2) | 1.1 | 1 (1-2) | 1.8 | 2 (1-2) |
| **Line 7_2_** | | | | | | | | | | | | |
| Tissue | **3 dpi** | | | | **7 dpi** | | | | **12 dpi** | | | |
|  | **D23580** | | **4/74** | | **D23580** | | **4/74** | | **D23580** | | **4/74** | |
|  | Mean | Median (range) | Mean | Median (range) | Mean | Median (range) | Mean | Median (range) | Mean | Median (range) | Mean | Median (range) |
| Caecum | 0.8 | 1 (0-1) | 1.4 | 2 (0-2) | 1 | 1 (0-2) | 1.2 | 1 (1-2) | 0.8 | 1 (0-1) | 1.5 | 1.5 (1-2) |
| Liver | 1.6 | 2 (1-2) | 1.4 | 1 (1-2) | 2 | 2 (2-2) | 1.6 | 2 (1-2) | 1.4 | 1 (1-2) | 2 | 2 (2-2) |
| Spleen | 1.8 | 2 (1-3) | 2 | 2 (1-3) | 1.6 | 2 (1-2) | 1.8 | 1.5 (1-3) | 1.2 | 1 (1-2) | 1.5 | 1.5 (1-2) |
| **Line Cb4** | | | | | | | | | | | | |
| **Tissue** | **3 dpi** | | | | **7 dpi** | | | | **12 dpi** | | | |
|  | **D23580** | | **4/74** | | **D23580** | | **4/74** | | **D23580** | | **4/74** | |
|  | Mean | Median (range) | Mean | Median (range) | Mean | Median (range) | Mean | Median (range) | Mean | Median (range) | Mean | Median (range) |
| **Caecum** | 1.6 | 1 (0-3) | 1 | 1 (0-2) | 1 | 1 (0-2) | 1.6 | 2 (1-2) | 1.1 | 1 (0-2) | 1.2 | 1 (0-2) |
| **Liver** | 1.6 | 2 (1-2) | 1.3 | 1 (0-2) | 1.6 | 2 (0-3) | 2.1 | 2 (1-4) | 1.3 | 1 (0-2) | 1.5 | 1.5 (0-4) |
| **Spleen** | 1.7 | 2 (1-2) | 1.7 | 2 (1-2) | 1.3 | 1 (1-2) | 1.6 | 2 (1-2) | 1.7 | 2 (1-3) | 1.8 | 2 (1-3) |
| **Line 15** | | | | | | | | | | | | |
| **Tissue** | **3 dpi** | | | | **7 dpi** | | | | **12 dpi** | | | |
|  | **D23580** | | **4/74** | | **D23580** | | **4/74** | | **D23580** | | **4/74** | |
|  | Mean | Median (range) | Mean | Median (range) | Mean | Median (range) | Mean | Median (range) | Mean | Median (range) | Mean | Median (range) |
| **Caecum** | 0.9 | 1 (0-1) | 1.7 | 2 (1-3) | 1.2 | 1 (1-2) | 1.9 | 2 (0-4) | 1 | 1 (1-1) | 1.3 | 1 (1-2) |
| **Liver** | 1.1 | 1 (0-2) | 1.6 | 2 (1-2) | 1.5 | 1.5 (1-2) | 1.6 | 2 (1-2) | 1.2 | 1 (0-2) | 1.3 | 1 (1-2) |
| **Spleen** | 0.6 | 1 (0-1) | 1.3 | 1 (1-2) | 1.2 | 1 (1-2) | 1.3 | 1 (1-3) | 1 | 1 (1-1) | 0.7 | 1 (0-1) |
