# Supplementary material for: The use of chicken and insect infection models to assess the virulence of African Salmonella Typhimurium ST313
Source: PLoS Negl Trop Dis. 2019 Jul 26;13(7):e0007540. doi: 10.1371/journal.pntd.0007540 (PMC6685681; doi:10.1371/journal.pntd.0007540)
Supplement: S8 Table — The qualitative assessment of the histopathological changes associated with infection at 3, 7 and 12 dpi was made by a veterinary pathologist. (DOCX) [file pntd.0007540.s008.docx]

|  |  | **Line 6_1_** | **Line 7_2_** | | **Line Cb4** |
| --- | --- | --- | --- | --- | --- |
| **Caecum** | **D23580** | Mild to moderate changes after 3 days that decline to mild after 7 and 12 days | Mild to moderate changes after 3 days that decline to mild after 7 and 12 days | | Mild to moderate changes after 3 days that decline to mild after 7 and 12 days |
|  | **4/74** | Moderate changes after 3 days that decline to mild after 7 and 12 days – slightly more severe than D23580 | Moderate changes after 3 days that decline to mild after 7 and 12 days – slightly more severe than D23580 | | Mild changes after 3 days, moderate after 7, decline to mild after 12 days – similar to D23580 |
| **Liver** | **D23580** | Mild changes after days 3, but more developed after day 7, declined by day 12 | Mild to moderate changes after days 3, but more developed after day 7, declined by day 12 | | Mild to moderate changes after days 3 and 7 days, declined slightly by day 12 |
|  | **4/74** | Moderate changes after day 3 but declining to mild after days 7 and 12. Early lesions more severe than D23580, but then similar | Mild to moderate changes after day 3, becoming more severe after 7 and 12. Similar to D23580580after 3 and 7 days, more severe after 12 | | Mild to moderate changes after day 3, moderate after day 7, declining slightly by day 12. Early lesions similar to D23580, but peak lesion at day 7 more severe |
| **Spleen** | **D23580** | Mild changes after day 3, progressing to mild to moderate after 7 and 12 | Mild to moderate changes after day 3, similar after 7, slightly declining after 12 | | Mild to moderate changes throughout |
|  | **4/74** | Mild to moderate after days 3, moderate after 7 then declining after 12. Slightly more severe than D23580 | Mild to moderate after days 3, declining after 7 and 12. Slightly more severe than D23580 | | Mild to moderate changes throughout – similar to D23580 |
|  |  | **Line W** | | **Line 15** | |
| **Caecum** | **D23580** | Mild to moderate changes after 3 days that decline to mild after 7 and 12 days | | Mild changes throughout | |
|  | **4/74** | Mild to moderate changes after 3 days that decline to mild after 7 and 12 days – similar to D23580 | | Mild to moderate changes, small peak at day 7. Slightly more severe than D23580 | |
| **Liver** | **D23580** | Mild to moderate changes after days 3 and 7, declined by day 12 | | Mild changes after days 3, but more developed after day 7, declined by day 12 | |
|  | **4/74** | Mild to moderate changes after day 3, but declining to mild after days 7 and 12 – similar to D23580 | | Mild to moderate changes after days 3 and 7, small decline by day 12. Slightly more severe than D23580 | |
| **Spleen** | **D23580** | Mild changes after day 3, progressing to mild to moderate after 7 then declining after 12 | | Mild changes after day 3, progressing to mild to moderate after 7 and 12 | |
|  | **4/74** | Mild after days 3, then mild to moderate after 7 and 12. Similar to D23580 after 3 and 7 days, slightly more severe after day 12 | | Mild to moderate after days 3, moderate after 7 then declining after 12. Slightly more severe than D23580 | |
