# Supplementary material for: The use of chicken and insect infection models to assess the virulence of African Salmonella Typhimurium ST313
Source: PLoS Negl Trop Dis. 2019 Jul 26;13(7):e0007540. doi: 10.1371/journal.pntd.0007540 (PMC6685681; doi:10.1371/journal.pntd.0007540)
Supplement: S9 Table — (DOCX) [file pntd.0007540.s009.docx]

| Residuals:  Min 1Q Median 3Q Max  -1.73240 -0.48137 -0.07034 0.48636 2.70750 | | | | | | | | | |
| --- | --- | --- | --- | --- | --- | --- | --- | --- | --- |
| Coefficients: | | | | | | | | | |
|  | Estimate | | Std. Error | | | t value | | Pr(>\|t\|) |  |
| (Intercept) | 1.22E+00 | | 8.97E-02 | | | 1.36E+01 | | 2.00E-16 | *** |
| Line = 6 | 5.34E-02 | | 9.63E-02 | | | 5.54E-01 | | 5.80E-01 |  |
| Line = 7 | 2.46E-01 | | 9.66E-02 | | | 2.55E+00 | | 1.10E-02 | * |
| Line = Cb4 | 2.43E-01 | | 8.90E-02 | | | 2.73E+00 | | 6.47E-03 | ** |
| Line = W | 2.75E-01 | | 8.97E-02 | | | 3.07E+00 | | 2.24E-03 | ** |
| Strain = D23580 | -2.22E-01 | | 5.92E-02 | | | -3.75E+00 | | 1.97E-04 | *** |
| Tissue = liver | 2.66E-01 | | 7.19E-02 | | | 3.70E+00 | | 2.43E-04 | *** |
| Tissue = spleen | 2.13E-01 | | 7.27E-02 | | | 2.93E+00 | | 3.54E-03 | ** |
| Timepoint = 7 dpi | 6.89E-02 | | 7.11E-02 | | | 9.69E-01 | | 3.33E-01 |  |
| Timepoint = 12 dpi | -1.99E-01 | | 7.33E-02 | | | -2.71E+00 | | 7.01E-03 | ** |
| Residual standard error: 0.6749 on 511 degrees of freedom  Multiple R-squared: 0.1011, Adjusted R-squared: 0.08524  F-statistic: 6.384 on 9 and 511 DF, p-value: 1.32e-08 | | | | | | | | | |
|  | | | | | | | | | |
| Response: pathology score  (0= no pathology, 4= maximum level of scored pathology) | | | | | | | | | |
|  | | Sum Sq | | Df | F value | | Pr(>F) | |  |
| (Intercept) | | 75.845 | | 1 | 1.68E+02 | | 2.20E-16 | | *** |
| Line | | 6.844 | | 4 | 3.78E+00 | | 4.85E-03 | | * |
| Strain | | 6.443 | | 1 | 1.42E+01 | | 1.81E-04 | | *** |
| Tissue | | 0.095 | | 2 | 1.05E-01 | | 9.01E-01 | |  |
| Timepoint | | 5.31 | | 2 | 5.87E+00 | | 3.03E-03 | | ** |
| Tissue * Timepoint | | 3.277 | | 4 | 1.81E+00 | | 1.26E-01 | |  |
| Residuals | | 229.509 | | 507 |  | |  | |  |

Significance levels: ‘***’ =0.001; ‘**’ =0.01, ‘*’ =0.05; ‘.’ =0.1; ‘ ’ =1
