# Supplementary material for: The use of chicken and insect infection models to assess the virulence of African Salmonella Typhimurium ST313
Source: PLoS Negl Trop Dis. 2019 Jul 26;13(7):e0007540. doi: 10.1371/journal.pntd.0007540 (PMC6685681; doi:10.1371/journal.pntd.0007540)
Supplement: S10 Table — (DOCX) [file pntd.0007540.s010.docx]

| Residuals:  Min 1Q Median 3Q Max  -1.6989 -0.4231 -0.118 0.5072 2.6864 | | | | | | | | | |
| --- | --- | --- | --- | --- | --- | --- | --- | --- | --- |
| Coefficients: | | | | | | | | | |
|  | Estimate | | Std. Error | | | t value | | Pr(>\|t\|) |  |
| (Intercept) | 1.18227 | | 0.1075 | | | 10.998 | | <2.00E-16 | *** |
| Line = 7 | 0.24175 | | 0.1008 | | | 2.398 | | 0.01706 | * |
| Line = Cb4 | 0.23799 | | 0.09288 | | | 2.562 | | 0.01087 | * |
| Strain = D23580 | -0.22606 | | 0.07908 | | | -2.858 | | 0.00455 | ** |
| Tissue = liver | 0.2786 | | 0.09606 | | | 2.9 | | 0.004 | ** |
| Tissue = spleen | 0.16351 | | 0.09699 | | | 1.686 | | 0.09285 | . |
| Timepoint = 7 dpi | 0.13133 | | 0.09477 | | | 1.386 | | 0.16681 |  |
| Timepoint = 12 dpi | -0.07797 | | 0.09778 | | | -0.797 | | 0.42583 |  |
| Residual standard error: 0.7042 on 310 degrees of freedom  Multiple R-squared: 0.08427, Adjusted R-squared: 0.06359  F-statistic: 4.075 on 7 and 310 DF, p-value: 2.70E-04 | | | | | | | | | |
|  | | | | | | | | | |
| Response: pathology score  (0= no pathology, 4= maximum level of scored pathology) | | | | | | | | | |
|  | | Sum Sq | | Df | F value | | Pr(>F) | |  |
| (Intercept) | | 41.537 | | 1 | 83.2376 | | <2.00E-16 | | *** |
| Line | | 4.131 | | 2 | 4.1388 | | 0.016844 | | * |
| Strain | | 4.047 | | 1 | 8.1093 | | 0.004702 | | ** |
| Tissue | | 0.998 | | 2 | 1.0003 | | 0.368977 | |  |
| Timepoint | | 0.783 | | 2 | 0.7849 | | 0.45706 | |  |
| Tissue * Timepoint | | 1.019 | | 4 | 0.5103 | | 0.728205 | |  |
| Residuals | | 152.7 | | 306 |  | |  | |  |

Significance levels: ‘***’ =0.001; ‘**’ =0.01, ‘*’ =0.05; ‘.’ =0.1; ‘ ’ =1
