# Supplementary material for: The use of chicken and insect infection models to assess the virulence of African Salmonella Typhimurium ST313
Source: PLoS Negl Trop Dis. 2019 Jul 26;13(7):e0007540. doi: 10.1371/journal.pntd.0007540 (PMC6685681; doi:10.1371/journal.pntd.0007540)
Supplement: S11 Table — (DOCX) [file pntd.0007540.s011.docx]

| Residuals:  Min 1Q Median 3Q Max  -1.35317 -0.45231 0.00438 0.42826 1.65312 | | | | | | | | | |
| --- | --- | --- | --- | --- | --- | --- | --- | --- | --- |
| Coefficients: | | | | | | | | | |
|  | Estimate | | Std. Error | | | t value | | Pr(>\|t\|) |  |
| (Intercept) | 1.34688 | | 0.11843 | | | 11.373 | | <2.00E-16 | *** |
| Line = W | 0.21857 | | 0.08861 | | | 2.467 | | 0.01449 | * |
| Strain = D23580 | -0.20597 | | 0.088 | | | -2.341 | | 0.02026 | * |
| Tissue = liver | 0.24638 | | 0.10637 | | | 2.316 | | 0.02158 | * |
| Tissue = spleen | 0.29496 | | 0.10803 | | | 2.73 | | 0.0069 | ** |
| Timepoint = 7 dpi | -0.03411 | | 0.10577 | | | -0.323 | | 0.74739 |  |
| Timepoint = 12 dpi | -0.39166 | | 0.10915 | | | -3.588 | | 0.00042 | *** |
| Residual standard error: 0.6248 on 196 degrees of freedom  Multiple R-squared: 0.1598, Adjusted R-squared: 0.1341  F-statistic: 6.213 on 6 and 196 DF, p-value: 5.46E-06 | | | | | | | | | |
|  | | | | | | | | | |
| Response: pathology score  (0= no pathology, 4= maximum level of scored pathology) | | | | | | | | | |
|  | | Sum Sq | | Df | F value | | Pr(>F) | |  |
| (Intercept) | | 52.758 | | 1 | 146.1561 | | <2.00E-16 | | *** |
| Line | | 2.496 | | 1 | 6.9156 | | 0.0092374 | | ** |
| Strain | | 2.175 | | 1 | 6.0248 | | 0.0149969 | | * |
| Tissue | | 1.298 | | 2 | 1.7975 | | 0.1684877 | |  |
| Timepoint | | 9.807 | | 2 | 13.5847 | | 3.03E-06 | | *** |
| Tissue * Timepoint | | 7.205 | | 4 | 4.9903 | | 0.0007552 | | *** |
| Residuals | | 69.306 | | 192 |  | |  | |  |

Significance levels: ‘***’ =0.001; ‘**’ =0.01, ‘*’ =0.05; ‘.’ =0.1; ‘ ’ =1
