# Supplementary material for: The use of chicken and insect infection models to assess the virulence of African Salmonella Typhimurium ST313
Source: PLoS Negl Trop Dis. 2019 Jul 26;13(7):e0007540. doi: 10.1371/journal.pntd.0007540 (PMC6685681; doi:10.1371/journal.pntd.0007540)
Supplement: S12 Table — (DOCX) [file pntd.0007540.s012.docx]

| **Virulence determinant** | **Strain** | **Virulence in chick embryos** | **Animal model in which attenuation of *S*. Typhimurium was observed:** | **Reference** |
| --- | --- | --- | --- | --- |
| SPI-1 | 4/74 Δ*hilD::frt* | Slightly attenuated | Chick, pig, cattle, mouse | 3 |
|  | D23580 Δ*hilC::frt* | Not attenuated | Mouse | 3 |
|  | 4/74 Δ*invA::frt* | Not attenuated | Chick, pig, cattle | 3 |
| PhoPQ regulon | 4/74 Δ*phoPQ::frt* | Not attenuated | Chick, cattle, mouse | 3 |
| SPI-2 | D23580 Δ*ssrAB::frt* | Not attenuated | Chick, pig, cattle, mouse | 3 |
| LPS | D23580 Δ*waaL::frt* | Not attenuated | Mouse | 4 |
|  | 4/74 Δ*waaG::frt* | Attenuated | Mouse | 4 |
|  | D23580 Δ*waaG::frt* | Attenuated | Mouse | 4 |
| Flagella | 4/74 Δ*fljAB::frt* Δ*fliC::frt* | Attenuated | Chick, pig, cattle, mouse | 3 |
| Motility | 4/74 Δ*motA::frt* | Not attenuated | Mouse | 5 |
