# Supplementary figures and images for: The use of chicken and insect infection models to assess the virulence of African Salmonella Typhimurium ST313
Source: PLoS Negl Trop Dis. 2019 Jul 26;13(7):e0007540. doi: 10.1371/journal.pntd.0007540 (PMC6685681; doi:10.1371/journal.pntd.0007540)

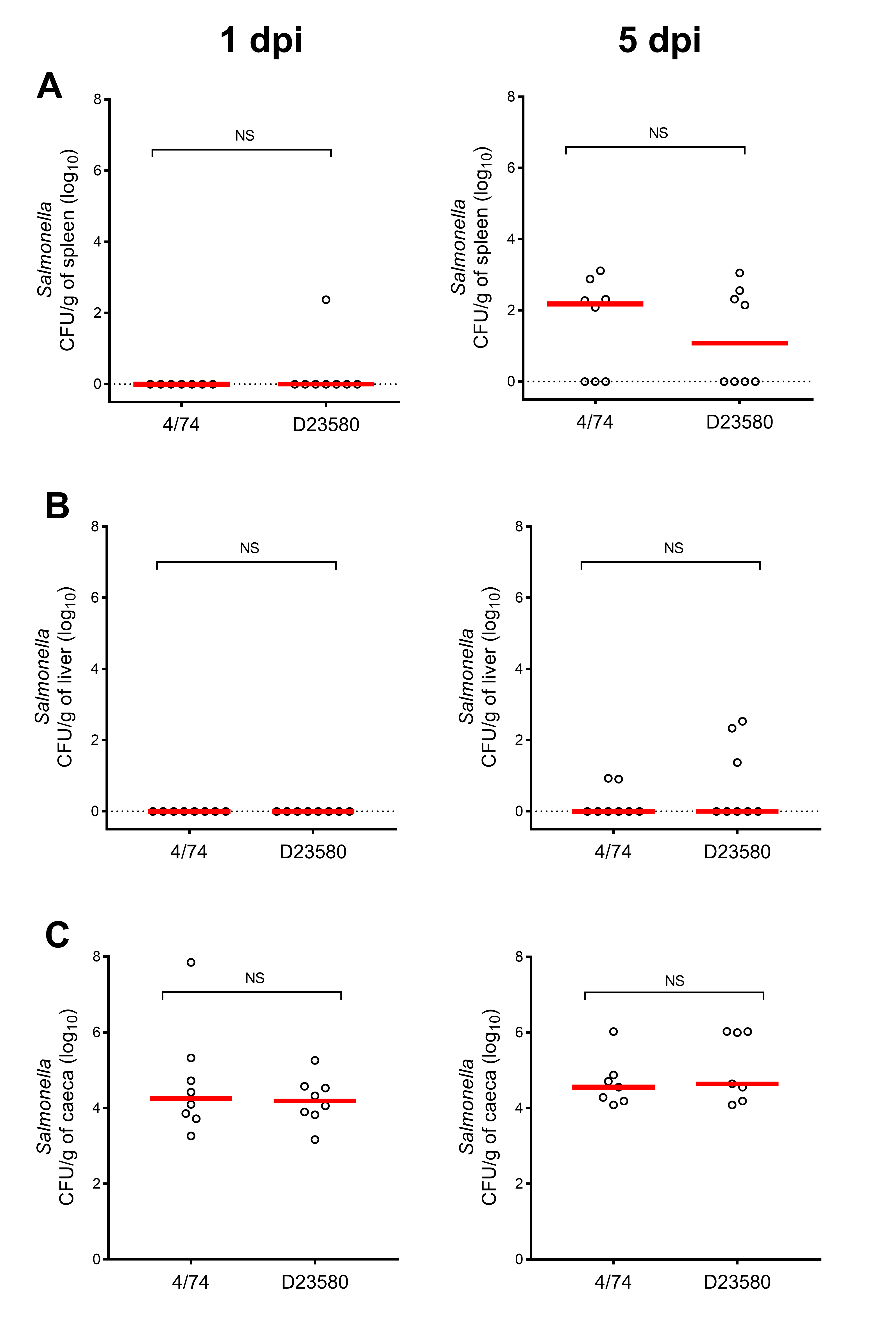

Supplement: S1 Fig — Viable counts (CFU/g) of S. Typhimurium 4/74 and D23580 in spleen (A), liver (B) and caecal content (C) of outbred chickens at 1 and 5 dpi. 14-day-old Lohman Brown Layers inoculated with 108 CFU. Counts are shown as individual birds with the bar representing the median value. Eight birds per group. Statistical comparison was made using a Mann-Whitney test. NS: not significant, P > 0.05. (TIF) [file pntd.0007540.s013.tif]

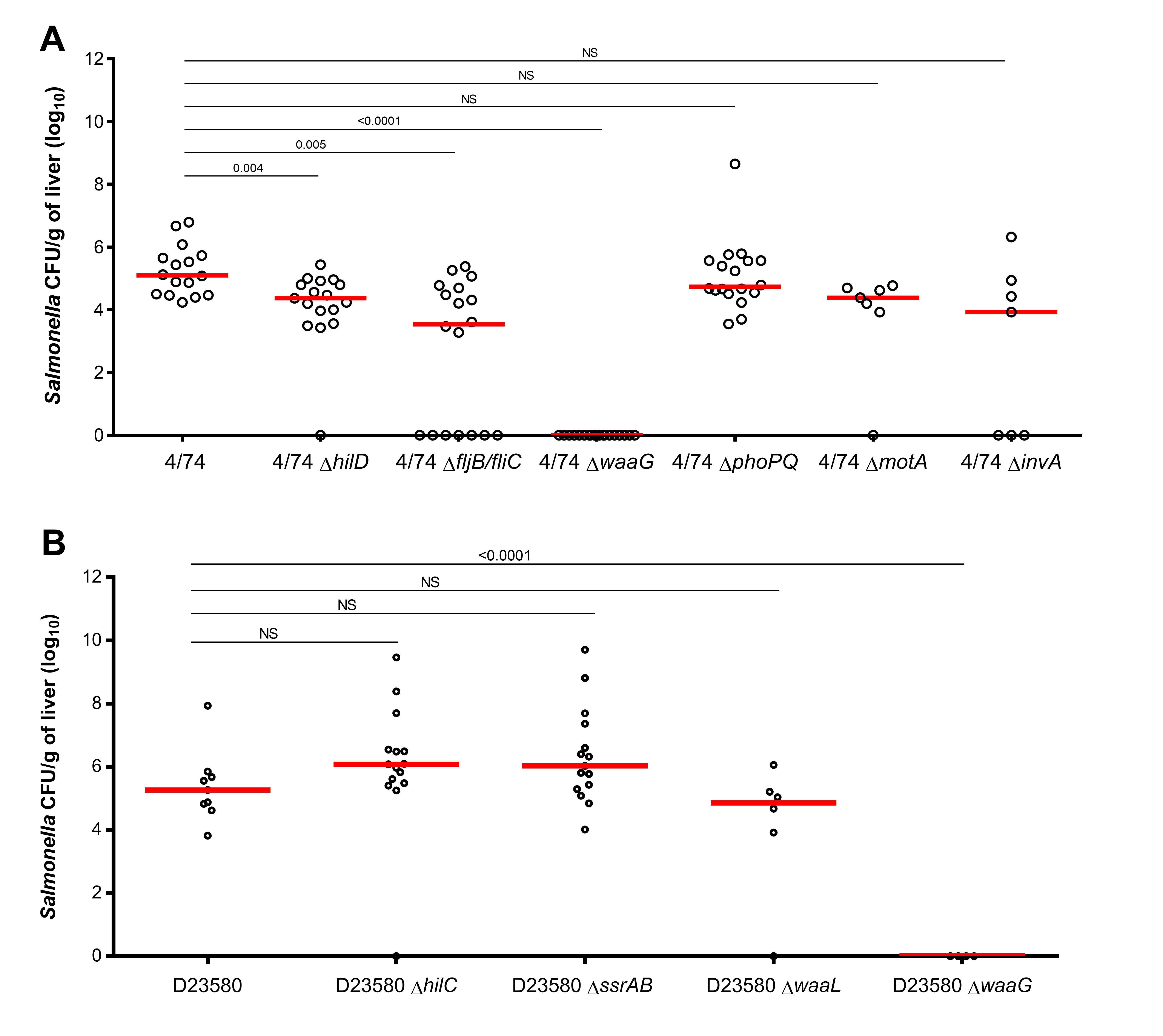

Supplement: S2 Fig — Attenuated mutants in the 4/74 (A) and D23580 (B) genetic background were tested. Bars represent the median values; circles represent individual embryos. Data from three independent experiments. Six embryos per group were used in each replicate. Individual groups (wild-type versus mutant) were compared using Mann-Whitney U test. (TIF) [file pntd.0007540.s014.tif]
