# Supplementary material for: The use of chicken and insect infection models to assess the virulence of African Salmonella Typhimurium ST313
Source: PLoS Negl Trop Dis. 2019 Jul 26;13(7):e0007540. doi: 10.1371/journal.pntd.0007540 (PMC6685681; doi:10.1371/journal.pntd.0007540)
Supplement: S1 Text — (DOCX) [file pntd.0007540.s016.docx]

**Salmonella virulence in the chick embryo model**

As *Salmonella* infection of chick embryos has not been well studied, we tested the ability of a number of mutants in important *Salmonella* virulence factors to infect the embryos. We used the wild-type  *S*. Typhimurium strains D23580 and 4/74 as positive controls and the respective Δ*rpoE* mutants (unable to mount a stress response and induce an immune response[1]) and *E. coli* K12 strains as negative controls. Both wild-type and attenuated strains were able to multiply within the allantoic cavity. However, while the *S*. Typhimurium Δ*rpoE* mutants and *E. coli* K12 did not leave the allantoic cavity, wild-type *S*. Typhimurium was found within the membrane that surrounds the allantoic cavity and in other egg compartments.

Next, we tested other mutants in reported *Salmonella* virulence factors*,* which have shown attenuation in other infection models (S12 Table). Intriguingly, SPI-1- and SPI-2-deficient strains remained virulent in the chicken embryo model while mutants in flagella and motility were just slightly attenuated, possibly due to reduced adhesion to the chorioallantoic membrane that surrounds the allantoic cavity. Strong attenuation was only seen in lipopolysaccharide (LPS) mutants, such as Δ*waaG* (S2 Fig). Bacterial infection of chick embryos induces migration of heterophils (avian equivalent of the mammalian neutrophil) which produces high quantities of antimicrobial peptides such as cathelicidins and β-defensins[2]. Since LPS mutants such as Δ*waaG* mutants have been reported to show increased susceptibility to antimicrobial peptides, we suggest that surviving these antimicrobial peptides might be the determining factor for virulence in this model.

**References**

1. Humphreys S, Stevenson A, Bacon A, Weinhardt AB, Roberts M. The Alternative Sigma Factor, ςE, Is Critically Important for the Virulence of *Salmonella* typhimurium. Infect Immun. 1999;67: 1560–1568.
2. Cuperus T, van Dijk A, Dwars RM, Haagsman HP. Localization and developmental expression of two chicken host defense peptides: cathelicidin-2 and avian β-defensin 9. Developmental & Comparative Immunology. 2016;61: 48–59. doi:10.1016/j.dci.2016.03.008.
3. Chaudhuri RR, Morgan E, Peters SE, Pleasance SJ, Hudson DL, Davies HM, et al. Comprehensive Assignment of Roles for *Salmonella* Typhimurium Genes in Intestinal Colonization of Food-Producing Animals. PLOS Genetics. 2013;9: e1003456. doi:10.1371/journal.pgen.1003456
4. Kong Q, Yang J, Liu Q, Alamuri P, Roland KL, Curtiss R. Effect of deletion of genes involved in lipopolysaccharide core and O-antigen synthesis on virulence and immunogenicity of *Salmonella* enterica serovar typhimurium. Infect Immun. 2011;79: 4227–4239. doi:10.1128/IAI.05398-11
5. Horstmann JA, Zschieschang E, Truschel T, de Diego J, Lunelli M, Rohde M, et al. Flagellin phase-dependent swimming on epithelial cell surfaces contributes to productive *Salmonella* gut colonisation. Cell Microbiol. 2017;19. doi:10.1111/cmi.12739
